# KLF15 cistromes reveal a hepatocyte pathway governing plasma corticosteroid transport and systemic inflammation

**DOI:** 10.1126/sciadv.abj2917

**Published:** 2022-03-09

**Authors:** Zhen Jiang, Selma Z. Elsarrag, Qiming Duan, Edward L. LaGory, Zhe Wang, Michael Alexanian, Sarah McMahon, Ingrid C. Rulifson, Sarah Winchester, Yi Wang, Christian Vaisse, Jonathan D. Brown, Mattia Quattrocelli, Charles Y. Lin, Saptarsi M. Haldar

**Affiliations:** 1 Amgen Research, South San Francisco, CA 94080, USA.; 2 Gladstone Institutes, San Francisco, CA 94158, USA.; 3 Department of Molecular and Human Genetics, Baylor College of Medicine, Houston, TX 77030, USA.; 4 Medical Scientist Training Program and Quantitative and Computational Biosciences Graduate Program, Baylor College of Medicine, Houston, TX 77030, USA.; 5 Biomedical Sciences Graduate Program, UCSF School of Medicine, San Francisco, CA 94143, USA.; 6 UCSF Diabetes Center and Department of Medicine, UCSF School of Medicine, San Francisco, CA 94143, USA.; 7 Division of Cardiovascular Medicine, Department of Medicine, Vanderbilt University Medical Center, Nashville, TN 37232, USA.; 8 Molecular Cardiovascular Biology Division, Heart Institute, Cincinnati Children’s Hospital Medical Center and Department of Pediatrics, University of Cincinnati College of Medicine, Cincinnati, OH 45229, USA.; 9 Kronos Bio Inc., Cambridge, MA 02142, USA.; 10 Cardiology Division, Department of Medicine, UCSF School of Medicine, San Francisco, CA 94143, USA.

## Abstract

Circulating corticosteroids orchestrate stress adaptation, including inhibition of inflammation. While pathways governing corticosteroid biosynthesis and intracellular signaling are well understood, less is known about mechanisms controlling plasma corticosteroid transport. Here, we show that hepatocyte KLF15 (Kruppel-like factor 15) controls plasma corticosteroid transport and inflammatory responses through direct transcriptional activation of *Serpina6*, which encodes corticosteroid-binding globulin (CBG). *Klf15*-deficient mice have profoundly low CBG, reduced plasma corticosteroid binding capacity, and heightened mortality during inflammatory stress. These defects are completely rescued by reconstituting CBG, supporting that KLF15 works primarily through CBG to control plasma corticosterone homeostasis. To understand transcriptional mechanisms, we generated the first KLF15 cistromes using newly engineered *Klf15*^3xFLAG^ mice. Unexpectedly, liver KLF15 is predominantly promoter enriched, including *Serpina6*, where it binds a palindromic GC-rich motif, opens chromatin, and transactivates genes with minimal associated direct gene repression. Overall, we provide critical mechanistic insight into KLF15 function and identify a hepatocyte-intrinsic transcriptional module that potently regulates systemic corticosteroid transport and inflammation.

## Teaser

The hepatocyte KLF15-CBG axis, revealed by KLF15 cistromes, controls systemic corticosteroid transport and inflammation.

## INTRODUCTION

In mammals, circulating corticosteroid hormones are synthesized by the adrenal gland and orchestrate adaptations to physiological stress across multiple organs (*1*). In target tissues, corticosteroids bind their cognate intracellular receptor [NR3C1; glucocorticoid receptor (GR)] (*2*, *3*) and trigger gene expression programs that mediate these adaptive physiological responses (*4*). A major function of corticosteroid hormones is to inhibit inflammatory signaling, acting as a rheostatic brake on exuberant immune activation (*5*, *6*). Inability to maintain corticosteroid bioavailability during disease-related stress can lead to unrestrained inflammatory activation, multiorgan failure, and death in humans (*7*). Corticosteroid drugs are also widely used anti-inflammatory agents, recently exemplified by their salutary effects in patients with severe SARS-CoV-2 infection (*8*). Hence, there has been intense interest in understanding the mechanisms regulating corticosteroid function in physiology and disease. The pathways governing endogenous corticosteroid biosynthesis (*9*) and degradation (*10*) are well understood. Likewise, the transcriptional mechanisms underlying the intracellular actions of corticosteroids have been extensively studied (*4*, *6*). In contrast, the regulatory pathways controlling systemic corticosteroid transport through the production of hormone-binding proteins—an equally essential determinant of active hormone function in vivo (*11*)—are less well understood.

Kruppel-like factor 15 (KLF15) is the most ancient member of the modern-day KLF family of zinc finger transcription factors (*12*). In mammals, KLF15 is expressed postnatally in liver, striated muscle, and fat, where it regulates key genes involved in nutrient and energy metabolism (*13*). Mice systemically deficient in KLF15 (*Klf15*^−/−^) are viable (*14*) but cannot adapt to metabolic stress, such as exercise (*15*) or fasting (*16*), phenotypes that are attributed to reduced expression of genes involved in gluconeogenesis, lipid metabolism, and branched-chain amino acid metabolism. More recent work has implicated KLF15 as a transcriptional repressor of hepatic genes controlling the metabolism of xenobiotics and endobiotics, suggesting that KLF15 deficiency might directly promote enhanced elimination of corticosteroids via increased expression of genes in this pathway (*17*). Here, we find that KLF15 directly transactivates *Serpina6* [which encodes corticosteroid-binding globulin (CBG)] in hepatocytes to potently control plasma corticosteroid transport in vivo and demonstrate that this KLF15-*Serpina6* transcriptional module plays a critical homeostatic role in the systemic response to inflammatory stress. In the course of these studies, we define the genome-wide binding patterns of KLF15 and provide important mechanistic insight into the function of this widely studied transcription factor.

## RESULTS

### KLF15 regulates *Serpina6* expression and plasma free corticosteroid concentration

**Fig. 1.**
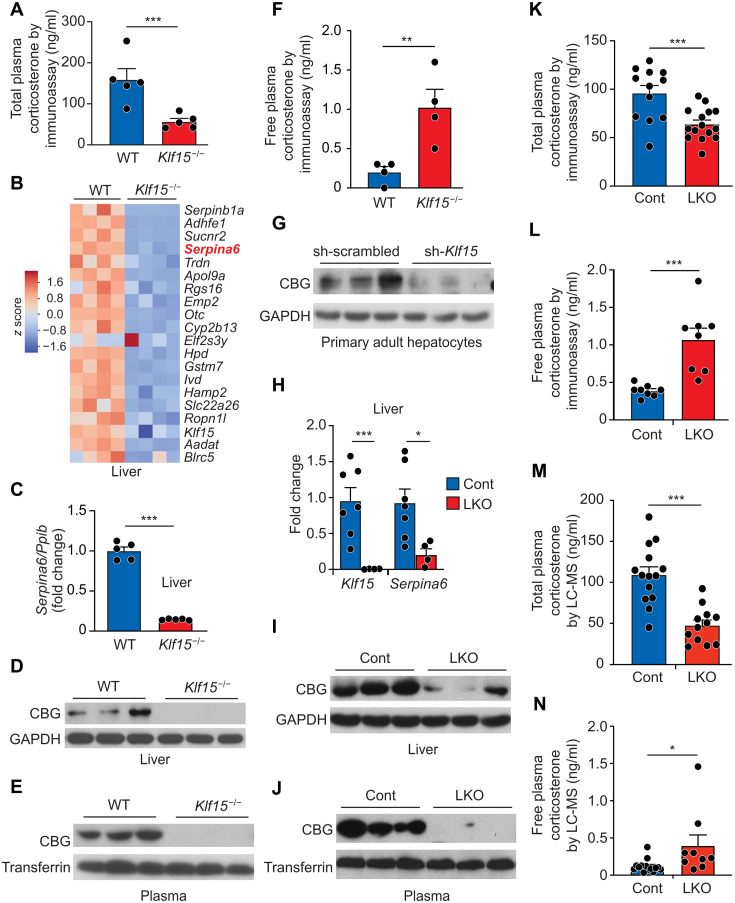
Hepatocyte KLF15 potently regulates SERPINA6/CBG expression and plasma corticosteroid binding capacity. (**A**) Plasma total corticosterone concentration in *Klf15*^−/−^ and *Klf15*^+/+^ mice (*N* = 5) quantified by radioimmunoassay. (**B**) Gene expression microarray data from livers of adult *Klf15*^−/−^ and *Klf15*^+/+^ mice (*N* = 4; from GSE7137). Heatmap of the top 20 most down-regulated genes in *Klf15*^−/−^ liver. *Serpina6* encodes CBG. (**C**) qRT-PCR from mouse liver tissue for *Serpina6* (normalized to *Ppib*; *N* = 5). (**D**) Western blot for CBG and GAPDH (loading control) from mouse liver tissue (*N* = 3; quantification provided in fig. S1F). (**E**) Western blot for CBG and transferrin (loading control) from mouse plasma (*N* = 3; quantification provided in fig. S1G). (**F**) Plasma free corticosterone concentration in *Klf15*^−/−^ and *Klf15*^+/+^ mice (*N* = 4) quantified by radioimmunoassay. (**G**) Western blot for CBG and GAPDH (loading control) in primary mouse hepatocytes infected with Ad-sh-KLF15 and sh-scrambled (*N* = 3; quantification provided in fig. S1I). (**H**) qRT-PCR for *Klf15* and *Serpina6* mRNA in the liver tissue of KLF15-LKO (*N* = 7) versus control (*Klf15*^flox/flox^; *N* = 4) mice. Data were normalized to *Ppib*. (**I**) Western blot for CBG and GAPDH (loading control) from mouse liver tissue (*N* = 3; quantification provided in fig. S1K). (**J**) Western blot for CBG and transferrin (loading control) from mouse plasma (*N* = 3; quantification provided in fig. S1L). (**K**) Total plasma corticosterone (*N* = 12 to 15) and (**L**) free plasma corticosterone (*N* = 8) concentration in plasma from LKO versus control mice quantified by radioimmunoassay at the National Institutes of Health MMPC at the University of California, Davis. (**M**) Total plasma corticosterone (*N* = 12 to 14) and (**N**) free plasma corticosterone (*N* = 9 to 13) concentration in plasma from LKO versus control mice quantified by liquid chromatography–mass spectrometry (LC-MS). Data are shown as means ± SEM. *P < 0.05, **P < 0.02, and ***P < 0.01 by two-tailed, unpaired *t* test.

Our study began with the unexpected observation that *Klf15*^−/−^ mice, despite having chronic hypoglycemia and substantially elevated plasma glucagon concentration (*16*), had inappropriately low plasma concentration of total corticosterone, the predominant circulating corticosteroid hormone in mice (Fig. 1A). We expected that plasma corticosterone, like glucagon, would also be elevated as part of a classic compensatory response to chronic hypoglycemia and metabolic stress. Initially, we hypothesized that the observed low plasma corticosterone concentration stemmed from a primary defect in corticosterone synthesis. However, we did not detect significant *Klf15* expression in the murine adrenal cortex (fig. S1A), the major site of de novo corticosteroid synthesis in mammals. In addition, expression of key enzymes in the corticosteroid biosynthetic pathway was not reduced in adrenal glands of *Klf15*^−/−^ mice (fig. S1B), and adrenal gland weight did not differ between genotypes (fig. S1C). The principal stimulus for corticosteroid biosynthesis by the adrenal cortex is the peptide hormone ACTH (adrenocorticotropic hormone), which is secreted by the anterior lobe of the pituitary gland. *Klf15*^−/−^ mice did not have decreased plasma ACTH concentration (fig. S1D), and we did not detect *Klf15* expression in the pituitary gland (fig. S1E). Collectively, these observations suggested that the low plasma total corticosterone concentration in *Klf15*^−/−^ mice could not be attributed to a defect in biosynthesis, prompting us to find another plausible molecular mechanism that could explain this hormone defect.

We analyzed transcriptomic profiles of several tissues from *Klf15*^−/−^ mice (*16*, *18*, *19*) and were intrigued by the 14-fold decreased expression of *Serpina6* in the liver of adult *Klf15*^−/−^ mice, which was the fourth most down-regulated transcript in this tissue (Fig. 1B). *Serpina6* encodes CBG, which is highly expressed by hepatocytes and abundantly secreted into the circulation, where it functions as the major carrier for circulating plasma corticosteroids (*11*, *20*, *21*). Pyrexia, acidosis, or site-specific cleavage of CBG by inflammatory proteases, such as neutrophil elastase, significantly reduces the corticosteroid binding affinity of CBG, events that are thought to promote local release of corticosteroids at sites of inflammation as a mechanism to homeostatically contain excessive inflammatory activation (*11*, *22*–*25*). As plasma CBG concentration is known to vary substantially in human physiology and disease (*26*–*29*), understanding the molecular mechanisms governing its abundance has been of significant interest (*30*–*33*). However, the direct transcriptional regulators and cis-regulatory elements that govern *Serpina6*/CBG expression in vivo, particularly those that act as critical “on switches” at the *Serpina6* locus, are not well characterized. Our transcriptomic data suggested that KLF15 might serve as a critical transcriptional activator of hepatocyte *Serpina6* expression and plasma CBG abundance in vivo.

We found that *Serpina6* mRNA and protein were profoundly reduced in livers of *Klf15*^−/−^ mice (Fig. 1, C and D, and fig. S1F). Furthermore, we detected a 90% decrease in plasma CBG concentration in *Klf15*^−/−^ mice (Fig. 1E and fig. S1G). *Klf15*^−/−^ mice also had elevated free plasma corticosterone concentration (Fig. 1F), a pathognomonic feature of reduced corticosteroid binding capacity and CBG deficiency. This elevation in free corticosterone excludes reduced hormone biosynthesis or enhanced hormone degradation as primary mechanisms underlying the abnormality in total corticosterone, because either of these potential mechanisms would result in low concentrations of both free and total corticosterone. Thus, our observation that free plasma corticosterone is actually three- to fourfold elevated in *Klf15*^−/−^ mice supports the hypothesis that KLF15 controls plasma corticosterone homeostasis primarily via inducing CBG and less via other putative mechanisms, such as inhibiting genes in the endobiotic biotransformation pathway, as has been previously proposed (*17*).

We next assessed whether KLF15 regulated CBG expression and function in a hepatocyte-autonomous manner. In primary cultured adult mouse hepatocytes, KLF15 overexpression induced *Serpina6/*CBG mRNA and protein (fig. S1H), while KLF15 knockdown with a short hairpin RNA (shRNA) decreased *Serpina6/*CBG mRNA and protein abundance (Fig. 1G and fig. S1I). Likewise, KLF15 knockdown in the human HepG2 cell line decreased *SERPINA6* (fig. S1J), confirming that this regulatory axis is conserved in human hepatocytes. As circulating cytokines and hormones can influence CBG abundance in vivo (*30*–*33*), the data from isolated primary hepatocytes in vitro confirm that gain or loss of function of KLF15 is sufficient to alter CBG expression in a hepatocyte-autonomous manner. To definitively establish a cell-autonomous role for hepatocyte KLF15 in vivo, we generated mice with hepatocyte-specific deletion of *Klf15* by crossing *Klf15*^flox/flox^ and *Albumin1*-Cre strains to generate *Albumin1*-Cre:*Klf15*^flox/flox^ mice (termed KLF15-LKO). We found that KLF15-LKO mice had decreased CBG in liver (Fig. 1, H and I, and fig. S1K), decreased plasma CBG (Fig. 1J and fig. S1L), decreased total plasma corticosterone concentration (Fig. 1K), and increased free plasma corticosterone concentration (Fig. 1L), mirroring the corticosteroid binding defect identified in *Klf15*^−/−^ mice (Fig. 1, A, E, and F), in *Serpina6*^−/−^ mice (*21*), and in humans harboring germline loss-of-function variants in *SERPINA6* (*34*–*37*). To confirm the findings of our radioimmunoassay-based measurements of plasma corticosterone, we quantified plasma corticosterone in an independent cohort of KLF15-LKO and control mice using a liquid chromatography–mass spectrometry (LC-MS) assay. The LC-MS data were consistent with our radioimmunoassay, confirming that KLF15-LKO mice had decreased total plasma corticosterone concentration and increased free plasma corticosterone compared to control mice (Fig. 1, M and N).

### Creation of *Klf15*^3xFLAG^ knock-in mouse enables detection of endogenous KLF15 protein

**Fig. 2.**
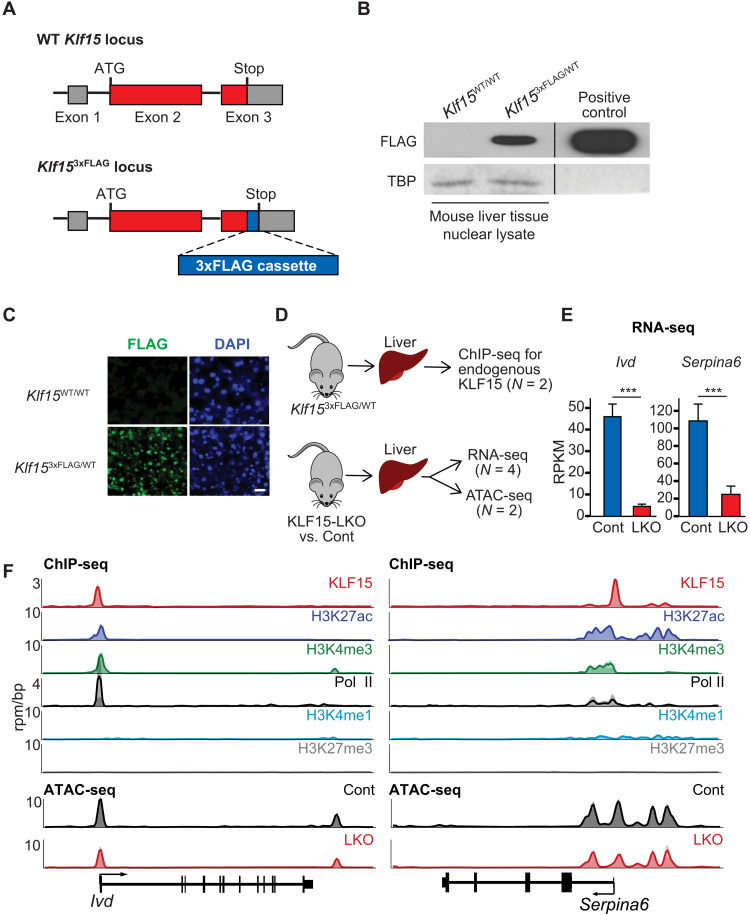
KLF15 ChIP-seq using a newly generated *Klf15*^3xFLAG^ knock-in mouse line. (**A**) Schematic of wild-type (WT) mouse *Klf15* genomic locus (top) and the genome-edited locus after insertion of a 3XFLAG epitope tag at the C terminus of endogenous KLF15 (bottom). (**B**) Western blot of mouse liver tissue nuclear protein from the indicated genotypes using FLAG antibody. TBP (TATA-binding protein) is the loading control. Positive control is the whole-cell lysate from 293 cells transfected with a mammalian expression plasmid encoding full-length mouse KLF15-3xFLAG fusion protein (pCDNA3.1-mKLF15-3xFLAG). Positive control is from the same immunoblot from a nonadjacent lane, designated by a vertical black bar. (**C**) FLAG immunofluorescence staining of liver tissue sections from adult *Klf15*^3xFLAG/WT^ mice and *Klf15*^WT/WT^ control mice. Scale bar, 20 μm. DAPI, 4′,6-diamidino-2-phenylindole. (**D**) Schematic of epigenomic assays performed on adult mouse liver. (**E**) Gene expression in units of reads per kilobase per million mapped reads (RPKM) for *Ivd* (isovaleryl-coenzyme A dehydrogenase) and *Serpina6* in adult mouse liver from the indicated genotypes. Data are shown as means ± SEM (*n* = 4). *Ivd* is a metabolic gene known to be regulated by KLF15 that serves as a positive control. ***P < 0.001 by exact test. (**F**) ChIP-seq (*n* = 2) and ATAC-seq (*n* = 2) gene tracks at the *Ivd* locus (left) and *Serpina6* locus (right) from adult mouse liver tissue. KLF15 signal is from FLAG ChIP-seq from *Klf15*^3xFLAG/+^ mice. Histone marks and RNA polymerase II (Pol II) enrichment are from ENCODE ChIP-seq data from adult liver tissue of WT mice. Individual replicates (*n* = 2) are plotted as translucent shapes, and the plotted line represents the mean signal over replicates.

As KLF15 deficiency causes robust reduction in CBG abundance, we hypothesized that *Serpina6* was a direct transcriptional target of KLF15 in adult hepatocytes. Despite the long-standing interest in KLF15 biology and extensive efforts in the field, there are currently no antibodies available that are capable of robustly and specifically detecting endogenous KLF15 with acceptable fidelity for chromatin immunoprecipitation (ChIP). Hence, the genome-wide binding sites of this intensely studied transcription factor remain unknown. To provide unbiased and whole-genome level insight into KLF15 mechanism of action, including at the *Serpina6* locus, we engineered a germline mouse strain harboring a 3x-FLAG epitope tag fused to the C terminus of endogenous KLF15 using Cas9-based genome editing of the endogenous locus (termed *Klf15*^3xFLAG^ allele; Fig. 2A). This engineered mouse strain enables mechanistic studies of endogenously expressed KLF15 and unbiased interrogation of KLF15 cistromes in the context of adult tissues in vivo. Mice harboring the *Klf15*^3xFLAG^ allele were viable and fertile in both the heterozygous and homozygous states (fig. S2A). Anti-FLAG immunoblots from the liver tissue of these mice unambiguously confirmed robust detection of a specific band of the expected size (Fig. 2B). FLAG immunofluorescence staining of mouse liver tissue also confirmed a signal for KLF15 (Fig. 2C). Notably, liver tissue from *Klf15*^3xFLAG/WT^ mice did not exhibit changes in expression of genes known to be sensitive to KLF15 abundance, including *Serpina6* (fig. S2B), supporting that the *Klf15*^3xFLAG^ allele did not perturb the normal transcriptional function of KLF15.

### Endogenous KLF15 regulates *Serpina6* by binding its proximal promoter

Using this new in vivo tool, we performed ChIP with anti-Flag antibody coupled to massively parallel DNA sequencing (ChIP-seq) in adult mouse liver tissue to determine genome-wide KLF15 enrichment. Concomitantly, we performed RNA sequencing (RNA-seq) and assay for transposase-accessible chromatin using sequencing (ATAC-seq) in the liver tissue from adult KLF15-LKO versus control mice to assess the relationship between locus-specific KLF15 enrichment, gene expression, and chromatin accessibility (schematized in Fig. 2D). We identified 7080 KLF15 binding sites across the genome (fig. S3A). Gene ontology analysis of genes proximal to KLF15 peaks showed enrichment for metabolic processes and related terms (fig. S3B), consistent with known pathways associated with KLF15 (e.g., amino acid catabolism, gluconeogenesis, urea cycle, and lipid metabolism) (*15*, *16*, *38*). We first focused on the *Ivd* (isovaleryl-coenzyme A dehydrogenase) and *Serpina6* loci, the former being a branched-chain amino acid metabolic gene known to be regulated by KLF15 (*16*). In our RNA-seq, both *Ivd* and *Serpina6* mRNA levels were reduced by approximately 80% in livers of KLF15-LKO mice (Fig. 2E). At both loci, we detected a discrete KLF15 binding site at the transcription start site (TSS), with overlapping peaks for RNA polymerase II (Pol II), the promoter-associated histone modifications H3K4me3 (histone 3 lysine 4 trimethyl) and H3K27ac (histone 3 lysine 27 acetyl), and accessible chromatin (Fig. 2F). In KLF15-LKO mice, we observed decreased chromatin accessibility at the *Ivd* and *Serpina6* promoters (Fig. 2F), where KLF15 normally binds. Together, these locus-specific observations support that KLF15 directly regulates *Serpina6* expression in the liver via binding at the proximal promoter.

### Genome-wide analysis reveals that liver KLF15 is predominantly promoter-bound and acts primarily as a transactivator

**Fig. 3.**
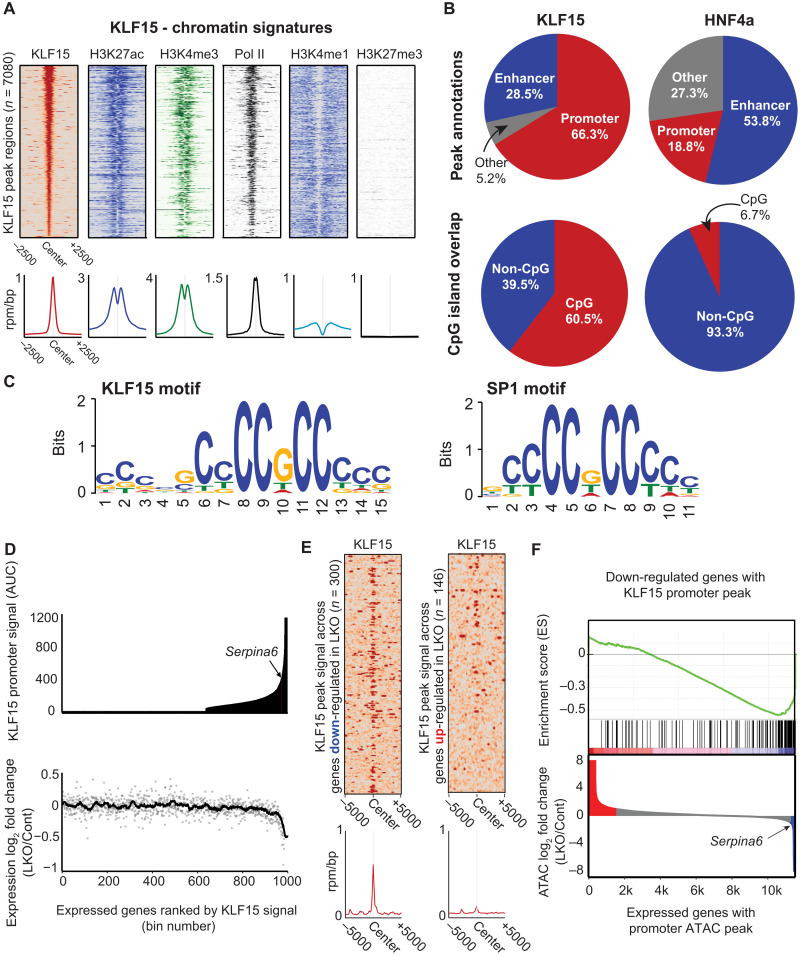
Genome-wide analyses reveal that liver KLF15 is predominantly promoter bound, where it opens the chromatin and functions as a transactivator. (**A**) Heatmaps of KLF15, H3K27ac, H3K4me3, RNA Pol II, H3K4me1, and H3K27ac ChIP-seq signal (rpm/bp) along 5-kb regions centered at KLF15 peaks. Regions are ordered on the basis of the KLF15 transcription factor peak signal (highest to lowest). Average signal profile (rpm/bp) is plotted below each heatmap. (**B**) Top row: Peak annotations for KLF15 (left) and HNF4a (right). Promoter peaks are defined as those falling within 2 kb of a known TSS, while enhancer peaks are defined as nonpromoter peaks overlapping a high-confidence region of H3K27ac. Bottom row: Proportion of overlap between KLF15 peaks (left) and HNF4a peaks (right) with known CpG islands. (**C**) Top motif identified from sequences within KLF15 peak regions (left; *E* = 7.6 × 10^−109^). Known SP1 motif is shown on the right. (**D**) Top: Median KLF15 ChIP-seq signal over TSSs for expressed genes subdivided into 1000 bins and rank ordered by KLF15 TSS signal. Bottom: Median log_2_ fold change (KLF15-LKO/control) for genes within each bin. (**E**) Heatmap of KLF15 ChIP-seq signal ±5 kb around the TSS of genes with decreased expression in KLF15-LKO (left) and increased in KLF-LKO (right). Average signal profile (rpm/bp) is plotted below each heatmap. (**F**) Top: Gene set enrichment analysis (GSEA) leading edge enrichment score for the set of genes down-regulated in KLF15-LKO and the set of genes with KLF15 promoter peak by ChIP-seq (*n* = 143). Bottom: Waterfall plot of total promoter ATAC-seq peak signal ranked by log_2_ fold change (KLF15-LKO/control).

We next quantified and characterized KLF15 DNA binding in the mouse liver at genome scale. As exemplified by the *Ivd* and *Serpina6* loci, we identified a consistent pattern of KLF15 enrichment at gene promoters, colocalizing with Pol II and H3K4me3 (Fig. 3A). KLF15 also colocalized with H3K27ac, which is present at both active promoter and enhancers (*39*), but colocalized minimally with H3K4me1 (histone 3 lysine 4 monomethyl), an enhancer-specific chromatin modification (Fig. 3A) (*39*). KLF15 did not colocalize with H3K27me3 (histone 3 lysine 27 trimethyl), a marker of heterochromatin and transcriptional repression (Fig. 3A) (*39*). In contrast, HNF4a, another nodal hepatocyte transcription factor (*40*), colocalized primarily with H3K27ac and H3K4me1 and exhibited less overlap with H3K4me3 and Pol II (fig. S3C). The distinct predilection of KLF15 to occupy promoter regions as compared to HNF4a is quantified via ChIP-seq peak annotations for representative chromatin marks (Fig. 3B, top) and overlap with promoter-enriched CpG islands (Fig. 3B, bottom). The distribution of peak distances to the closest genes is notably different between KLF15 and HNF4a (fig. S3D), with a higher proportion of KLF15 peaks (70%) falling within 5 kb of the TSS of a gene (versus 40% for HNF4a). De novo motif enrichment analysis revealed that the KLF15 consensus DNA binding motif was a specific element that was GC-rich and palindromic, nearly identical to the consensus motif for SP1 (Fig. 3C), a ubiquitously expressed transcription factor known to be highly enriched in promoter CpG islands across the genome (*41*).

To further dissect KLF15-dependent gene regulatory mechanisms, we analyzed changes in gene expression between KLF15-LKO and control mouse livers. We confirmed that hepatocyte-specific deletion of *Klf15* strongly decreased its own transcript levels in our RNA-seq data. Among all detectable KLF family transcription factors, only *Klf10* also exhibited a significant change in gene expression in the livers of KLF15-LKO mice (fig. S4A). We next investigated whether loss of KLF15 had a broader impact on the expression of its interactome, which was defined using experimentally validated and predicted interactions from the STRING database (fig. S4B and table S1), and found no KLF15 interactors as differentially expressed. These data suggest that hepatocyte *Klf15* deficiency does not broadly affect the expression levels of KLF family genes or the predicted KLF15 interactome.

We next defined the set of transcripts that are differentially expressed in KLF15-LKO versus control mice (fig. S4C) and analyzed the relationship between differential expression and local KLF15 binding (fig. S4D). Using gene set enrichment analysis (GSEA), we identified enrichment for metabolic pathways in the set of transcripts significantly down-regulated in LKO mice (fig. S4E, top), consistent with the enrichment for metabolic genes bound by KLF15 in our ChIP-seq data (fig. S3B). Inflammation-related transcripts were up-regulated in KLF15-LKO mice (fig. S4E, bottom); however, the set of KLF15-bound genes was not enriched for these inflammatory targets (fig. S3B). We more closely examined the relationship between KLF15 promoter occupancy and the directional change in expression of the proximate assigned genes in KLF15-LKO mice (Fig. 3D). Consistent with a direct regulatory role, we found a positive correlation between KLF15 occupancy signal and decline in gene expression in KLF15-LKO tissue, with the largest decline in gene expression observed among expressed genes with the highest levels of KLF15 at their TSS (Fig. 3D). In contrast to down-regulated genes, genes up-regulated in KLF15-LKO showed little to no KLF15 enrichment signal (Fig. 3E and fig. S4D), suggesting that the up-regulation of these genes occurs via indirect mechanisms that do not involve KLF15 binding. These data suggest that, under physiological conditions, hepatocyte KLF15 predominantly binds gene promoters and directly mediates gene induction but does not generally function as a direct transcriptional repressor. As hepatocyte KLF15 regulates CBG abundance, we also assessed whether the GR/NR3C1 transcriptional program was affected in the liver of KLF15-LKO mice. *Nr3c1* expression itself was unchanged in KLF15-LKO versus control livers (fig. S5A). Next, we defined the directly bound targets of NR3C1 by curating a published dataset in which NR3C1 ChIP-seq was performed in the mouse liver (*42*). When we ranked genes by proximal NR3C1 binding occupancy, we observed no relationship between NR3C1 binding and gene expression changes upon *Klf15* deletion in the liver (fig. S5A). In addition, we compared the total promoter KLF15 signal to the total distal HNF4a signal among all expressed genes and found that many genes down-regulated in KLF15-LKO, including *Serpina6*, exhibit high levels of proximal KLF15 without any distal HNF4a (fig. S5B). This indicates that a specific subset of adult hepatocyte genes, including *Serpina6*, are more dependent on regulation by proximal promoter KLF15 binding than they are by tissue-specific distal enhancers or other transcription factor programs.

Locus-specific analysis of our ATAC-seq data showed that KLF15 deficiency led to decreased chromatin accessibility at the *Ivd* and *Serpina6* promoters, where KLF15 is normally bound (Fig. 2F). We therefore hypothesized that KLF15 may generally be necessary to maintain open chromatin at its binding sites genome wide. To determine the change in ATAC-seq peaks between KLF15-LKO and control livers, we ranked the consensus set of ATAC-seq peaks across both conditions by signal and examined the change in rank of all promoter proximal ATAC-seq peaks. We found that the highest ranked promoter proximal ATAC-seq peaks were also among those with the highest KLF15 signal and that these peaks typically exhibited a decrease in both rank and signal in KLF15-LKO mice (fig. S5, C and D). In addition, the set of genes with the highest decline in ATAC-seq signal upon KLF15-LKO showed enrichment for down-regulated genes that have a promoter proximal KLF15 peak (Fig. 3F). Last, we sought to identify other transcription factors putatively associated with KLF15 binding sites. We found that motifs for SP1, E2F4, and ZFX, which have been independently identified as being promoter associated (*43*, *44*), frequently co-occur with sites of KLF15 enrichment (fig. S6). In contrast, HNF4a is associated with a distinct set of hepatocyte regulatory transcription factors, including COT2 (NR2F2), PPARA, and PPARG (fig. S6) (*40*). Together, the epigenomic data in Figs. 2 and 3 demonstrate that, under physiological conditions, endogenous liver KLF15 has a distinct predilection for binding a specific palindromic GC-rich consensus element on gene promoters, where it is required for maintaining open chromatin and activating the expression of a subset of tissue-specific gene targets such as *Serpina6*.

### KLF15 works through CBG to regulate plasma corticosterone and the homeostatic response to inflammatory stress

**Fig. 4.**
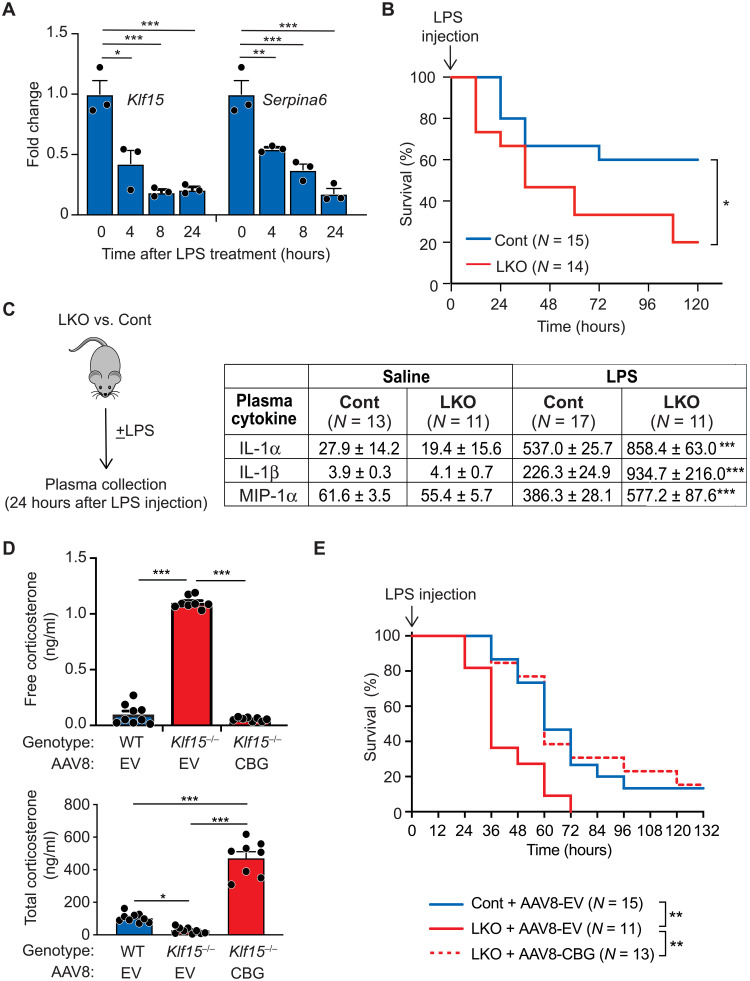
Hepatocyte KLF15 controls plasma corticosteroid bioavailability and inflammatory homeostasis via regulation of CBG in vivo. (**A**) qRT-PCR analysis of *Klf15* and *Serpina6* mRNA expression in WT mouse primary hepatocytes stimulated with LPS (200 ng/ml). *N* = 3. *P < 0.05, **P < 0.02, and ***P < 0.01 by one-way ANOVA test followed by Bonferroni’s multiple comparison correction. (**B**) Survival of mice after intraperitoneal injection of LPS (15 μg/g of body weight LPS; *N* = 15). *P < 0.05 by log-rank test. (**C**) Plasma cytokine concentration in KLF15-LKO and control mice 24 hours after injection with LPS (12 μg/g of body weight) or saline. ***P < 0.01 for the effect of KLF15-LKO genotype status on LPS response using two-way ANOVA followed by Tukey’s multiple comparison test. A full panel of measured cytokines is provided in table S1. (**D**) Free and total corticosterone concentration in plasma of adult mice of the indicated genotypes (quantified by radioimmunoassay), measured 6 weeks after tail vein injection with indicated AAV8. *N* = 8 to 9. *P < 0.05 and ***P < 0.01 by two-way ANOVA followed by Tukey’s multiple comparison test. (**E**) Mice of indicated genotypes were given AAV8-CBG or AAV8-EV via tail vein injection. Six weeks after administration of AAV8, mice were given LPS (15 μg/g of body weight, intraperitoneally) and assessed for survival (*N* = 11 to 15). **P < 0.02 versus LKO + AAV8-EV by log-rank test. Data are shown as means ± SEM.

We next asked whether this KLF15-CBG transcriptional module that we identified regulates the homeostatic response to inflammatory stress, a process in which KLF15 has not been previously implicated. While hepatocyte KLF15 is an important regulator of gluconeogenesis (*16*), *Serpina6*^−/−^ mice have normal blood glucose in the fed and fasted states (*21*), suggesting that CBG does not play a critical role in gluconeogenesis in vivo. We therefore interrogated the function of KLF15-CBG axis in the context of inflammatory stress because CBG is known to act as a robust physiological brake on inflammation (*11*, *21*–*25*). In adult hepatocytes, both *Klf15* and *Serpina6* mRNAs were coordinately down-regulated after inflammatory stimulation with lipopolysaccharide (LPS) (Fig. 4A), indicating that this module is under dynamic control during inflammatory stress. Despite having increased plasma free cortisol concentration (Fig. 1L), KLF15-LKO mice challenged with LPS had increased mortality compared to controls (Fig. 4B). In an independent cohort of KLF15-LKO and control mice, we assayed plasma cytokines 24 hours after LPS challenge to capture inflammatory states preceding the differences in mortality. KLF15-LKO mice had no differences in cytokine levels at baseline but did demonstrate elevated plasma concentrations of several cytokines after LPS stimulation, such as interleukin-1α (IL-1α), IL-1β, and macrophage inflammatory protein-1α (Fig. 4C and table S2), signifying an inability to restrain the inflammatory response.

To definitively establish that the corticosteroid defects observed in *Klf15*-deficient mice were caused by decreased CBG expression, we performed in vivo rescue experiments to restore CBG expression in the livers and plasma of *Klf15*-deficient mice. We generated adeno-associated virus serotype 8 (AAV8) harboring a complementary DNA (cDNA) encoding full-length murine CBG and first validated this reagent in *Klf15*^−/−^ mice, whose highly penetrant defects in CBG expression and corticosteroid binding capacity allow for efficient physiological validation. AAV8-CBG administration via tail vein injection rescued CBG protein abundance in the livers and plasma of *Klf15*^−/−^ mice (fig. S7, A and B) and completely rescued the defect in corticosteroid binding capacity in the plasma of *Klf15*^−/−^ mice, with a correction of free and total plasma corticosterone (Fig. 4D). Thus, even in the setting of global *Klf15* deficiency, restoring the expression of this single plasma protein corrected the defects in circulating corticosterone concentration. These data support the conclusion that KLF15 controls plasma corticosterone homeostasis primarily via regulating CBG production. We next hypothesized that the higher mortality seen in KLF15-LKO mice in the setting of LPS challenge (Fig. 4B) was also due to decreased CBG expression. We observed that AAV8-CBG treatment fully improved mortality in KLF15-LKO mice back to the same level as in control mice (Fig. 4E). Collectively, these studies demonstrate that KLF15 governs plasma corticosteroid hormone transport and the systemic response to inflammatory stress through robust regulation of CBG production by hepatocytes.

## DISCUSSION

**Fig. 5.**
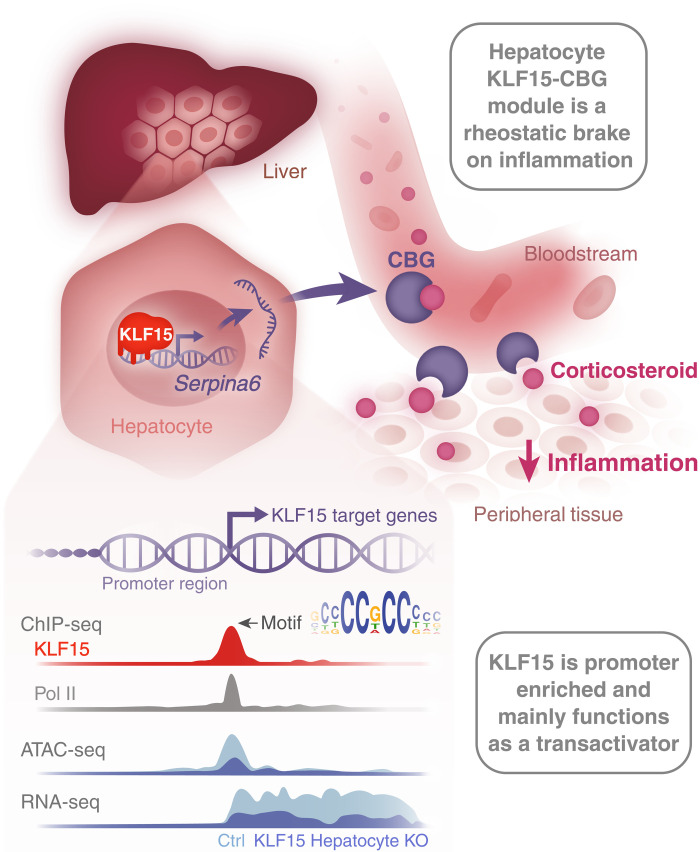
Summary diagram. In hepatocytes, the zinc finger transcription factor KLF15 directly binds the *Seprina6* proximal promoter and transactivates *Serpina6* mRNA expression, leading to the synthesis of CBG protein. CBG is abundantly secreted by hepatocytes into the bloodstream, where it regulates free versus total corticosteroid hormone concentration and dynamically releases bound corticosteroids at sites of inflammation as a mechanism to rheostatically suppress local inflammatory activation. Deletion of *Klf15* in mouse hepatocytes in vivo leads to profound reduction of plasma CBG concentration, decreased plasma corticosterone binding capacity, and increased mortality during acute inflammatory challenge. These phenotypes are completely rescued by reconstituting CBG expression in the livers and plasma of *Klf15*-deficient mice in vivo. Using the newly generated *Klf15*^3xFLAG^ mouse strain described in this study, ChIP-seq for endogenous KLF15 was performed in adult mouse liver tissue and integrated with RNA-seq and ATAC-seq from liver tissue of mice deficient versus sufficient in hepatocyte KLF15. In the liver, KLF15 was found to be predominantly enriched at gene promoters, where it binds a palidromic GC-rich consensus element, maintains open chromatin, and transactivates proximal target genes.

Our study provides key insights into steroid hormone physiology, inflammatory homeostasis, and KLF15 mechanism of action that were previously unknown (summary diagram provided in Fig. 5). As corticosteroids are master regulators of physiology, mammals have evolved robust mechanisms to spatiotemporally control the bioactivity of this potent hormone. For example, corticosteroid synthesis in the adrenal gland is tightly regulated via a feedback loop involving the hypothalamus, pituitary gland, and adrenal cortex (*6*). Likewise, inside target cells, the function of ligand-activated GR is precisely controlled via protein interactions, posttranslational modifications, and DNA sequence variants in a highly context-specific manner (*4*, *6*). In contrast to these well-studied aspects of corticosteroid biology, the signaling pathways that dynamically control plasma corticosteroid hormone transport are not as well defined, despite the central importance of this process in physiology and disease. Our study demonstrates that KLF15 regulates plasma corticosteroid transport and thereby inflammatory homeostasis via direct and specific transcriptional activation of CBG production by hepatocytes in vivo. While KLF15 is known to play an important role in nutrient metabolism (*13*), our discovery of the hepatic KLF15-CBG axis now implicates this transcription factor as a nodal regulator of inflammation, providing an important link between these two fundamental homeostatic processes.

### Unbiased assessment of genome-wide KLF15 enrichment reveals new mechanistic insights

To date, studies of KLF15 transcriptional function have empirically focused on individual gene promoters. KLF15 ChIP-seq has not been previously reported because of a lack of tools to appropriately probe endogenous KLF15 protein. Therefore, the extent to which KLF15 functions through binding at promoters versus distal elements or directly participates in gene activation versus gene repression has remained unclear because of a lack of unbiased genome-wide maps of endogenous KLF15 enrichment. Our epigenomic studies reveal that promoter-based regulation of target gene transactivation by KLF15 is not unique to the *Serpina6* locus but is actually a general feature of KLF15-dependent gene control in the liver. We also find that genes that are up-regulated during KLF15 deficiency, many of which have been considered to be transcriptionally repressed by KLF15, generally lack KLF15 enrichment at the locus. This finding suggests that the primary genomic function of KLF15 is to bind promoters and transactivate proximal targets, while the repressive effects of KLF15 generally do not involve DNA binding and thus are more likely to involve indirect mechanisms. KLF15’s pattern of genome-wide promoter enrichment on specific, palindromic GC-rich consensus elements, which are highly similar to SP1 binding motifs, is consistent with prior phylogenetic studies that suggest that KLF15 evolved from ancient SP1-like transcription factors to form the ancestral clade of the modern-day KLF family (*12*). We note that other KLF family members with highly tissue-specific roles, such as KLF1 in erythropoiesis, have been shown to bind predominantly at distal regulatory elements (*45*). We have also shown that HNF4a, a nodal regulator of hepatocyte gene expression, mainly enriches at nonpromoter elements (Fig. 3B). This predominance of promoter-based gene induction by liver KLF15 that we have observed in our genome-wide analyses is an unusual mode of action for a transcription factor that is expressed in terminally differentiated cells and dynamically controls a cluster of tissue-specific genes that govern adult mammalian physiology. In the future, when endogenous KLF15 cistromes are generated from other tissues using the *Klf15*^3xFLAG^ mouse strain, it will be informative to see how the KLF15 genome-wide distribution pattern in extrahepatic tissues compares to that observed here in the adult mouse liver.

We note that depletion of hepatocyte KLF15 only partially reduces chromatin accessibility at these KLF15-bound targets, suggesting that other factors can maintain chromatin accessibility at these regulatory elements in its absence. Although the decrease in chromatin accessibility at these loci was partial, mRNA levels of KLF15-bound genes such as *Serpina6* and *Ivd* were substantially reduced in our RNA-seq. This suggests that KLF15 might regulate additional steps in transcription that are downstream of local chromatin opening, such as Pol II initiation or elongation. Follow-up studies to explore the consequences of KLF15 deficiency on Pol II dynamics or nascent transcription would be interesting areas of future investigation. In addition, it will be informative to gain a deeper mechanistic understanding of KLF15-dependent gene control through the discovery and functional characterization of its protein interactome. While our epigenomic analyses and STRING database interrogation have provided some early insights into putative KLF15 co-regulators and protein interaction networks, the creation of the *Klf15*^3xFLAG^ knock-in mouse strain reported in this study provides future opportunities to discover the interactome of endogenously expressed KLF15 using unbiased proteomic approaches.

### The KLF-CBG module in the context of other pathways involved in CBG and plasma corticosterone homeostasis

As plasma CBG concentration dynamically varies in physiology and disease, there has been interest in understanding the precise mechanisms regulating its abundance. For example, plasma CBG concentration has been shown to be decreased in patients with infection and various inflammatory states (*26*–*29*), and low plasma CBG concentration associates with poor outcomes in patients with sepsis (*46*) and chronic liver disease (*47*). Inflammatory stimulation by cytokines such as IL-6 can suppress CBG abundance in HepG2 cells in vitro through a mechanism that does not involve changes in *SERPINA6* transcription (*30*), while thyroid hormone has been shown to increase CBG production via effects on *Serpina6* mRNA stability (*32*). Corticosteroid hormones can decrease CBG production in adult mammals (*31*, *32*) through a mechanism that may involve indirect repression of the *Serpina6* promoter by the GR (*48*). We find that KLF15 enriches at the *Serpina6* promoter in hepatocytes and that KLF15 overexpression or deficiency results in robust *Serpina6*/CBG induction or depletion, respectively, in a hepatocyte-autonomous manner. These data implicate KLF15 as an essential “on switch” that directly controls physiological CBG abundance in vivo. In the context of organismal development and postnatal maturation, corticosteroid hormones have been shown to induce *Serpina6*/CBG in the immature mammalian liver (*33*, *49*). As KLF15 itself is a direct transcriptional target of the GR (*18*, *19*, *50*), our work raises the possibility that the GR-KLF15 axis may be important in establishing CBG expression during postnatal maturation in mammals or may have a role in counterbalancing pathways that negatively regulate CBG production in the adult liver. Notably, the human sex hormone–binding globulin (*SHBG*) gene promoter has been shown to be activated by HNF4a and repressed by COUP-TF, implicating competition between these two factors for critical cis-regulatory elements in the locus as a mechanism of regulation (*51*). While we have clearly identified KLF15 as an essential on switch for *Serpina6*, it is possible that KLF15 deficiency also sets the stage for an additional “off switch” to exert repressive effects on *Serpina6*, analogous to what occurs at the *SHBG* locus. We highlight that two recent genome-wide association studies reported that common variants in the human *SERPINA6* locus were the top associations with plasma cortisol concentration in healthy human participants (*52*, *53*), raising the possibility that some of these human *SERPINA6* DNA variants may influence the ability of KLF15 to regulate CBG production in hepatocytes. In future studies, it will be interesting to explore how KLF15 mechanistically intersects with other signaling axes, cis-regulatory variants, and trans-regulatory factors to fine-tune CBG abundance in the contexts of postnatal maturation, adult physiology, and disease.

A recent study has suggested that KLF15 regulates total plasma corticosterone abundance via repressive effects on genes involved in metabolism, biotransformation, and elimination of endobiotics (*17*). The authors observed that mice deficient in hepatocyte *Klf15* have reduced total plasma corticosterone concentration, a finding that they attribute to increased expression of hepatic genes involved in corticosteroid hormone biotransformation and degradation. However, this study did not directly measure steroid hormone biotransformation (e.g., glucuronidation, hydroxylation, and sulfation) and subsequent elimination in *Klf15*-deficient cells or animals. Furthermore, no direct molecular mechanism is shown by which endobiotic metabolism genes are up-regulated in *Klf15*-deficient animals, and no rescue of endogenous corticosterone homeostasis is demonstrated by specific inhibition of the endobiotic metabolism machinery. By contrast, our current study shows that the low total plasma corticosterone in *Klf15*-deficient mice is accompanied by a three- to fourfold elevation in free plasma corticosterone (Fig. 1), a phenotype that can only be explained by CBG deficiency or impaired CBG function and not by enhanced degradation via the endobiotic metabolism pathway (enhanced corticosterone degradation/elimination would lead to a decrease in both free and total plasma corticosterone). This singular finding of elevated free plasma corticosterone in *Klf15*^−/−^ and KLF15-LKO mice calls into question whether enhanced hormone degradation/elimination is a primary mechanism by which KLF15 controls plasma corticosterone levels. Furthermore, in our current study, we completely rescue the abnormalities in both total and free plasma corticosterone concentration via reconstitution of plasma CBG in *Klf15*-deficient mice (Fig. 4), proving that the KLF15-CBG module is the principal mechanism of regulating plasma corticosterone in vivo. The previously published study also reported that that *Klf15*-deficient mice exhibit increased concentration of corticosterone in the urine (*17*). We note that *Serpina6*^−/−^ mice have been shown to have decreased plasma corticosterone half-life and increased corticosterone clearance (*21*), suggesting that the increased urinary corticosterone concentration seen in *Klf15-*deficient mice might be a consequence of reduced CBG expression. In addition to these physiological observations, our unbiased genome-wide assessment of KLF15 binding demonstrates that KLF15 is robustly enriched at promoters of genes whose expression is decreased in *Klf15*-deficient mice but does not generally enrich at genes that are up-regulated in the setting of *Klf15* deficiency (Fig. 3). This finding suggests that KLF15-dependent gene transactivation is direct (as occurs for *Serpina6*), while most of the putative repressive effects of KLF15 occur indirectly. Therefore, our data support the conclusion that many of the genes in the endiobiotic metabolism pathway are not direct targets of KLF15, suggesting that much of this gene up-regulation that occurs in KLF15-deficient livers may arise through secondary or indirect effects.

### The KLF15-CBG module is a physiological brake on systemic inflammation

Overall, our data are consistent with the hepatocyte KLF15-CBG module functioning as a physiological brake on systemic inflammation. Decreased activity of the hepatocyte KLF15-CBG module, as occurs during inflammatory stimulation (Fig. 4A), might be a mechanism that serves to augment inflammatory tone during early phases of infection or tissue injury. However, our findings identify that excessive suppression of the KLF15-CBG module can be maladaptive, leading to unbridled inflammatory activation and decompensation during sepsis, despite the presence of significantly increased free corticosterone concentration in bulk plasma. Our in vivo rescue experiments using AAV8-CBG, an intervention that increases corticosteroid binding capacity in bulk plasma and improves survival during experimental sepsis, support the evolving concept that adequate plasma CBG abundance is required for an optimal homeostatic response to inflammatory stress (*11*). This work suggests that excessive suppression of the KLF15-CBG axis may exacerbate illness, while strategies to boost CBG abundance could have a therapeutic benefit in certain settings.

## MATERIALS AND METHODS

### Study approval

All protocols concerning animal use were approved by the Institutional Animal Care and Use Committees (IACUC) at the University of California, San Francisco and conducted in strict accordance with the National Institutes of Health Guide for the Care and Use of Laboratory Animals, eighth edition. Mice were housed in a temperature- and humidity-controlled pathogen-free facility with 12-hour light/dark cycle and libitum access to water and standard laboratory rodent chow.

### Statistical analysis

Statistical analysis of genome sequencing data is described in a separate section that follows. All other statistical analysis was performed using GraphPad Prism (version 8). For survival analysis of KLF15-LKO mice, statistical analysis was performed using a log-rank test. For cytokine measurement, two-way analysis of variance (ANOVA) followed by Tukey’s multiple comparison test was applied to determine the effect of genotype on cytokine induction after LPS injection. For the down-regulation of *Klf15* and *Serpina6* in primary hepatocytes after LPS exposure (Fig. 4A), a one-way ANOVA test followed by Bonferroni’s multiple comparison test was used. For experiments assessing CBG protein abundance and plasma corticosterone concentration after rescue with AAV8-mSerpina6, a two-way ANOVA test followed by Tukey’s multiple comparison test was used. Statistical tests involving single comparisons between two experimental groups were determined using *t* test for unpaired samples. All values are expressed as means ± SEM, and individual data points are displayed wherever possible.

### Genetically modified mouse models

*Klf15*^−/−^ mice have been previously described (*14*). In these mice, a nuclear-localized *Lacz* expression cassette has been inserted into exon 2 of the endogenous *Klf15* locus to create a null allele and to also allow for detection of endogenous *Klf15* expression via assaying beta-galactosidase expression or activity. To generate mice with hepatocyte-specific *Klf15* deletion, *Alb1*-Cre^+/+^ mice [B6.Cg-Tg(Alb1-cre)21Mgn/J; the Jackson Laboratory, catalog no. 003574] were crossed with *Klf15*^flox/flox^ mice (*54*) to generate mice with hepatocyte-specific *Klf15* deletion (*Alb1*-Cre/*Klf15*^flox/flox^; termed KLF15-LKO mice). The control mice for KLF15-LKO mice are of genotype *Klf15*^flox/flox^. All mice were in a pure C57Bl/6 background, and our experiments were performed in male mice aged 14 to 20 weeks. All experiments were performed using littermate controls.

To generate mice harboring a *Klf15*^3xFLAG^ allele in the germ line, we used CRISPR-Cas9–based genome editing of mouse blastocysts (which were in a pure C57Bl/6 background) using direct injection of purified Cas9, guide RNA, and single-stranded DNA repair template, as previously described (*55*). Guide RNA and repair template were designed according to methods described by the laboratory of F. Zhang (*56*). The guide RNA (5′-CGGCCACUGCGCUCAGUUGA-3′) was designed to target the region immediately 5′ to the stop codon in exon 3 of the endogenous *Klf15* locus. The DNA repair template (5′-ACACATCAAAGTGCATCGCTTCCCACGAAGCAGCCGCGCAGTACGCGCCATCAACGGAGGCGGTGGAGCCGACTACAAGGACCACGACGGCGACTACAAGGACCACGACATCGACTACAAGGACGACGACGACAAGGGGCCTGTTTGAGCGCAGTGGCCGCCCTTCCCTCCCCCAGCTCCACGTTTTGTTTTTAAATGCA-3′) encodes a cassette with a short linker sequence followed by a 3xFLAG tag and stop codon. Approximately 50–base pair (bp) flanking homology arms are present on both sides of the 3xFLAG cassette (*57*). Following the final asparagine residue of mouse KLF15, this strategy fuses the following amino acid sequence to the C terminus: GGGGADYKDHDGDYKDHDIDYKDDDDKGPV*. After blastocyst injection and implantation, the F_0_ (mosaic) offspring were screened for the presence of the 3xFLAG cassette using polymerase chain reaction (PCR) amplification of tail genomic DNA, TA cloning, and DNA sequencing. Three founder lines that had high levels of mosaicism were crossed with male C57BL/6 mice from the Jackson Laboratory (catalog no. 000664) to generate F_1_ offspring, which were screened for germline transmission by PCR and sequencing of TA-cloned PCR amplicons from tail genomic DNA. Of these lines, we chose one line that was confirmed by PCR, sequencing of genomic DNA, and detection of the KLF15-3xFLAG fusion protein by FLAG Western blot. All *Klf15*^3xFLAG^ mice were in a pure C57Bl/6 background, and our experiments were performed in male mice aged 12 to 16 weeks. Genotyping primers used in fig. S2A are as follows: 5′-CGCTCAGATGAGTTGTCAAGG-3′ (forward) and 5′-GTGGCTCTGGGTCATACCT-3′ (reverse).

### Plasma corticosterone, ACTH, and aldosterone measurement by radioimmunoassay

Mice were euthanized, after which blood was immediately collected through the abdominal aorta (approximately 0.7 ml) and transferred into EP tubes that contained 500 mM EDTA (final concentration of EDTA was 1%). The tubes were then centrifuged at 1500*g* for 15 min at 4°C to separate plasma from blood cells. This plasma sample was used for the measurement of total corticosterone concentration. To measure free plasma corticosterone concentration, we used an aliquot of plasma and separated the protein-bound fraction by ultracentrifugation using a Centrifree ultrafiltration device (Millipore, 4104). Briefly, plasma was transferred into the ultrafiltration tubes and centrifuged at 2000*g* for 30 min. The ultrafiltrated and non-ultrafiltrated plasma were assayed for free or total corticosterone, respectively, by the Mouse Metabolic Phenotyping Center (MMPC) at the University of California, Davis using standardized radioimmunoassay. Non-ultrafiltrated plasma was also assayed for ACTH and aldosterone concentration by the Vanderbilt MMPC Hormone Assay and Analytical Services Core using standardized radioimmunoassays. For plasma corticosterone measurements, blood was taken from mice at 4:00 p.m. to coincide with the time of daily peak plasma corticosterone in mice.

### Plasma corticosterone measurement by LC-MS

A total of 20 μl of EDTA-plasma was used for total corticosterone measurement, and 150 to 200 μl of ultrafiltrated plasma were used for free corticosterone measurement in LC-MS experiments. Plasma corticosterone extraction was performed on the basis of previous reports with some modifications (*58*). First, samples were diluted to 200 μl in 0.2 M acetate buffer (pH 5.2) and then with 1.3 ml of acetate buffer plus 0.07% ascorbic acid and 5 ng of the internal standard, corticosterone-d4 (Sigma-Aldrich, 802905). Corticosterone was extracted from samples using an Oasis HLB μElution plate (Waters, 186008052) as follows: The SPE plate was preconditioned with 1 ml of methanol, followed by 2 ml of water. Samples were loaded onto the plate and then washed with 2 ml of water before elution with 1 ml of methanol. Methanol eluate was evaporated to dryness under a stream of nitrogen, and extracts were resuspended in 1:1 mobile phase A (water plus 0.1 mM ammonium fluoride):mobile phase B (methanol plus 0.1 mM ammonium fluoride) for analysis. Samples were analyzed using a Thermo TSQ Quantiva mass spectrometer coupled with a Thermo Ultimate 3000 ultrahigh-performance liquid chromatography (UPLC) system. Separation was performed by applying a binary gradient to an Acquity UPLC BEH C18 column (Waters, 186005604) using an LC-MS method adapted from previous reports (*59*). The gradient was initiated at 10% mobile phase B from 0 to 0.5 min before increasing to 30% mobile phase B between 0.5 and 1 min and then to 98% mobile phase B between 1 and 6 min. The gradient was maintained at 98% mobile phase B from 6 to 6.5 min before returning to 10% mobile phase B between 6.5 and 7 min and maintained at 10% mobile phase B from 7 to 8 min. Flow was maintained at 0.3 ml/min throughout the method, and column temperature was maintained at 50°C. Corticosterone and corticosterone-d4 levels were measured using selected reaction monitoring of the precursor/product ion combinations 347/121 mass/charge ratio (*m*/*z*) (collision energy of 24.63 V) and 351/121 *m*/*z* (collision energy of 25.56 V), respectively. Standard curves were prepared in acetate buffer using purified corticosterone standard and extracted as described above. Free and total corticosterone plasma concentrations were calculated using the ratio of corticosterone peak area/corticosterone-d4 peak area for each sample.

### LacZ staining

The adrenal gland was dissected from adult *Klf15*^−/−^ and *Klf15*^+/+^ mice and processed into 10-μm-thick cryosections on microscope slides. Slides were fixed for 5 min in 0.2% (w/v) glutaraldehyde [containing 0.1 M sodium phosphate buffer (pH 7.3), 5 mM EGTA, and 3 mM MgCl_2_]. Slides were washed three times in wash buffer [0.1 M sodium phosphate buffer (pH 7.3), 2 mM MgCl_2_, and 0.02% NP-40]. Each section was covered with LacZ Staining Solution [X-galactosidase (1 mg/ml; Invitrogen, 15520018), 5 mM potassium hexacyanoferrate(II) trihydrate (Sigma-Aldrich, 60279), and 5 mM potassium ferricyanide (Sigma-Aldrich, 702587)], dissolved in wash buffer, and incubated in the dark at 37°C overnight. Afterward, slides were washed three times for 5 min each in wash buffer followed by a 2-min wash in phosphate-buffered saline (PBS). Next, sides were dehydrated through sequential washes in increasing concentrations of ethanol and cleared with xylene (Thermo Fisher Scientific, 23-044-192). Slides were mounted with coverslips using VECTASHIELD hard-set antifade mounting medium (Vector Laboratories, H-1400). Images were then captured using a light microscope.

### Immunofluorescent staining

Liver, hypothalamus, and pituitary gland were dissected from adult mice and processed into 10-μm cryosections on microscope slides. Slides were washed twice for 7 min each in wash buffer (PBS and Tween 0.2%) followed by blocking sections with blocking buffer [10% goat serum, 0.3% Triton, 1.1% NaCl, 0.75% tris base, 1% bovine serum albumin (BSA), 1.8% l-lysine, and 0.04% azide (pH 7.4)] for 1 hour at room temperature. Slides were then washed twice for 7 min each in wash buffer. Primary antibody {anti–beta-galactosidase (1:200; Promega, Z3781) in antibody buffer [1.125% NaCl, 0.75% tris base, 1% BSA, 1.8% l-lysine, and 0.04% azide in water (pH 7.4)]} was then added onto sections and incubated for 2 days at 4°C. Subsequently, slides were washed in wash buffer three times for 10 min each followed by a 1-hour incubation at room temperature with secondary antibody [Alexa Fluor goat anti-mouse IgG (H + L) 488, 1:500, in antibody buffer] and 10-min incubation with 4′,6-diamidino-2-phenylindole at room temperature and mounted with Fluor gel (EMS, 17985-11). Images were then captured with a Keyence fluorescence microscope. This analysis was replicated in at least three independent samples.

### Culture of cell lines

293 cells and HepG2 cells were purchased from the American Type Culture Collection. They were routinely maintained in Eagle’s minimal essential medium (EMEM) with 10% fetal bovine serum. Transfection was performed with transfection reagent Fugene 6 (Promega, E2691) according to the manufacturer’s instructions. Adenovirus for the expression of full-length KLF15 (Ad-KLF15) and shRNA against KLF15 (Ad-sh*Klf15*) were produced and purified by Welgen Inc. (Worcester, MA) and have been previously described (*60*). For KLF15 overexpression, Ad-KLF15 was applied to media at a final concentration of 1 × 10^9^ VG per well in a six-well plate for 24 hours. For KLF15 knockdown, Ad-sh*Klf15* was applied to media at a final concentration of 1 × 10^9^ VG per well in a six-well plate. For LPS stimulation, cells were incubated with LPS (Sigma-Aldrich, L4525) in the media at a concentration of 200 ng/ml for the indicated times.

### Primary hepatocyte culture

Primary mouse hepatocytes were isolated from the liver of 6- to 8-week-old male C57BL/6 mice from the Jackson Laboratory. The isolation protocol was from the UCLA Center for Iron Disorders (www.iron.med.ucla.edu/protocol-primary-hepatocyte-isolation.html) with minor modifications. Liver was perfused with perfusion medium (Gibco, 17701-038) through the portal vein with an outlet at the inferior vena cava, followed by digestion with prewarmed liver digest medium (Gibco, 17703-034). Digested liver was then removed and placed in a cell culture plate that was filled with cold washing medium. Tissue was torn into finer pieces with forceps, shaken in the medium quickly to further dissociate, and poured through a 70-μm cell strainer. Hepatocytes were centrifuged at 80*g* for 5 min and washed with wash medium twice. Washed cells were resuspended with 10 to 20 ml of plating medium (Gibco, A1217601) containing supplement (Gibco, CM3000), after which cells were counted. Hepatocytes were then seeded and cultured in 0.1% gelatin-coated plates. Plates were placed in a 37°C incubator with 5% CO_2_ and 95% relative humidity for 3 hours, followed by one wash to remove dead cells. Medium was then replaced with maintenance medium (Gibco, A1217601) containing supplement (Gibco, CM4000). Medium was changed the next day, and experiments were initiated within 24 hours, as cells can typically be maintained in culture for approximately 4 days.

### RNA extraction and qRT-PCR

For RNA isolation from cells, total RNA was isolated using the High Pure RNA Isolation Kit (Roche, 11828665001) according to the manufacturer’s instructions. For RNA isolation from mouse tissues, tissue samples were disrupted/homogenized in TRIzol (Thermo Fisher Scientific) in a TissueLyser (QIAGEN) using stainless steel beads (QIAGEN) at 30 Hz for a total of 4 min. Total RNA was isolated using an Aurum Total RNA Fatty and Fibrous Tissue kit (Bio-Rad, 7326870). The purified RNA was then reverse-transcribed using the iScript Reverse Transcription Supermix for RT-qPCR (Bio-Rad, 1708841) according to the manufacturer’s instructions. Quantitative reverse transcription PCR (qRT-PCR) was performed with the TaqMan method (using the Roche Universal Probe Library System). A list of qRT-PCR primers and TaqMan probes are provided in table S3. Relative expression was calculated using the 2ΔΔCt method with normalization to Ppib/*Cyclophilin-b* expression in mouse or *GAPDH* expression in human.

### Western blotting

To extract total protein from cultured cells, cells were lysed in radioimmunoprecipitation assay (RIPA) buffer (Sigma-Aldrich, R0278) supplemented with protease inhibitor (Roche, catalog no. 4693132001) tablets. To extract total protein from liver tissue, the tissue was homogenized with a Dounce homogenizer and lysed in RIPA buffer supplemented with protease inhibitor. Lysed cells and tissues were centrifuged to remove nuclei and insoluble material. Nuclear protein was isolated using the NE-PER Nuclear and Cytoplasmic Extraction Reagents (Thermo Fisher Scientific, 78833) according to the manufacturer’s instructions. For each sample, 20 to 60 μg of protein extracts were subjected to Western analysis using SDS–polyacrylamide gel electrophoresis. For CBG Western blots, we used CBG antibody (Abcam, ab107368) for Fig. 1 and fig. S1. During the course of our studies, Abcam discontinued this particular CBG antibody. We therefore validated and used CBG antibody (US Biological, 139925) for all CBG Western blots appearing after Fig. 1 and fig. S1. Other primary antibodies used were FLAG antibody (Sigma-Aldrich, F1804), TATA-binding protein antibody (Abcam, Ab818), transferrin antibody (Santa Cruz Biotechnology, sc-52256), and glyceraldehyde phosphate dehydrogenase (GAPDH) antibody (Cell Signaling Technology, 2118S). Secondary horseradish peroxidase–conjugated antibodies and ECL detection system (Amersham, RPN2106) were applied, and signal was captured on standard autoradiographic film. As our loading controls were technically challenging or difficult to quantify after stripping and reprobing our Western blots for CBG and KLF15-3xFLAG, we ran loading controls on separate lanes or gels using a master lysate for each experimental sample that was preboiled in sample buffer at a fixed concentration of total protein (therefore, both the total protein and lysate volume loaded in each lane were the same). Densitometric quantification was performed using ImageJ. Given the very low CBG abundance in *Klf15*-deficient tissues, higher exposures than what is shown in the figures were required for densitometric quantification for certain Western blots.

### LPS-induced sepsis in mice

Mice were injected intraperitoneally with a single dose of LPS or normal saline control. LPS was obtained from Sigma-Aldrich (catalog no. L4524, lot no. 107M4048V). Doses used were 12 μg (for cytokine measurement) or 15 μg (for survival study) of LPS per gram of body weight. For cytokine measurement, LPS was injected at 10 a.m., and plasma was collected 24 hours after injection. Plasma cytokine concentration was measured by Eve Technologies using the Mouse Cytokine Array/Chemokine Array 31-Plex (MD31). For the survival studies, mice were observed every 12 hours after LPS injection. We recorded the number of mice that died or reached the standard prespecified criteria for humane euthanasia in our IACUC protocol. For LPS challenge in the setting of AAV8 administration, mice were injected with 5 × 10^11^ VG of AAV8-mSerpina6 or empty virus control (EV) via tail vein. Six weeks after tail vein injection with virus, mice were injected intraperitoneally with 15 μg of LPS per gram of body weight, and survival was recorded as above.

### Adenovirus and AAV construction

Adenovirus for the expression of full-length KLF15 (Ad-KLF15) and shRNA against KLF15 (Ad-sh*Klf15*) were produced and purified by Welgen Inc. (Worcester, MA) and have been previously described (*60*). For shRNA against mouse KLF15, the hairpin sequence 5′-GCGGTAAGATGTACATCAAACGTGTGCTGTCCGT TTGGTGTACATCTTGCTGC-3′ (loop sequence is underlined) was subcloned into the pEQ adenoviral shRNA vector (Welgen Inc.). Recombinant adenoviruses for sh-*Klf15* and sh-scrambled were amplified and purified by Welgen Inc. (*60*). Our recombinant AAV8 vector was designed to express a full-length mouse *Serpina6* cDNA under the control of the EF1a promoter. cDNA for full-length mouse *Serpina6* was cloned by Genewiz into an AAV vector called pAAV9-8434-DM vector (the number 9 is an internal catalog number for the plasmid and does not reflect the AAV serotype of the virus), which was then packaged and purified into high-titer AAV8 particles by SignaGen Laboratories. Detailed maps of the AAV8-EF1a-mSerpina6 construct are available upon request. Mice were injected with 5 × 10^11^ VG of AAV8-EF1a-mSerpina6 or EV control via tail vein, and mice were characterized 6 weeks after tail vein injection.

### Chromatin immunoprecipitation sequencing

ChIP was performed from mouse liver tissue as previously described (*38*). Liver tissue (∼300 mg) was freshly excised from adult male KLF15^3xFLAG/WT^ mice. The ChIP-seq replicates reported in our study are derived from two separate pieces of liver tissue that came from the same mouse. The liver tissue was minced, crushed, and fixed with 1.1% (w/v) formaldehyde for 10 min. For chromatin preparation, fixed tissue was homogenized in lysis buffer 1 [50 mM Hepes (pH 7.5), 140 mM NaCl, 1 mM EDTA, 10% glycerol, 0.5% NP-40, 0.25% Triton X-100, and protease inhibitor cocktail] using a Dounce homogenizer and then rotated at 4°C for 10 min. The pellet was spun down and resuspended in lysis buffer 2 [10 mM tris-HCl (pH 8.0), 200 mM NaCl, 1 mM EDTA, 0.5 mM EGTA, and protease inhibitor cocktail] for 10 min at 4°C. The pellet was spun down and resuspended in 1 ml of sonication buffer [50 mM Hepes (pH 7.5), 140 mM NaCl, 1 mM EDTA, 1 mM EGTA, 1% Triton X-100, 0.1% Na-deoxycholate, 0.1% SDS, and protease inhibitor cocktail]. The sonication buffer with chromatin was then sheared on a BioRuptor (Diagenode, Bioruptor Plus, UCD-300), with samples continuously cooled at 4°C. The BioRuptor settings used were 30 s on and 30 s off with the output setting on “high” for a total of 12 cycles. The sonicated chromatin was immunoprecipitated overnight at 4°C using 5 μg of anti-FLAG (Sigma-Aldrich, F1804) monoclonal antibody–bound Dynabeads (Invitrogen, 10004D), followed by extensive washing and elution. Input and immunoprecipitated chromatin were then reverse cross-linked followed by purification of genomic DNA, which was then subjected to library preparation and next-generation sequencing. Purified DNA was quantified using Qubit fluorometric analysis and assessed for quality using Agilent Bioanalyzer Nano DNA Chip. Library preparation was performed using the NEBNext Ultra II DNA Library Prep Kit from Illumina (New England Biolabs, E7645). Libraries were sequenced on a NextSeq 500/550 at the Genomics Core of Gladstone Institutes (single-end, 75 bp, >40 million reads per sample).

### RNA sequencing

Total RNA was extracted from liver tissues of KLF15-LKO mice and *Klf15*^flox/flox^ control mice by using the Aurum Total RNA Fatty and Fibrous Tissue kit (Bio-Rad, 7326870) according to the manufacturer’s instructions. RNA-seq libraries were prepared with the Ovation Solo RNA-Seq kit (NuGEN). The final libraries were analyzed by Agilent Bioanalyzer Nano RNA Chip and quantified by KAPA qPCR. The library was then sequenced on an Illumina HiSeq 2500 at the Genomics Core of Gladstone Institutes.

### Assay for transposase-accessible chromatin using sequencing

Library preparation and ATAC-seq were performed as previously described (*61*). Primary hepatocytes were isolated from liver tissues of KLF15-LKO mice and *Klf15*^flox/flox^ control mice as described above. For each sample, 50,000 cells were pelleted after washing once with 1 ml of PBS. Cell pellets were then resuspended in 50 μl of cold lysis buffer [10 mM tris-HCl (pH 7.4), 10 mM NaCl, 3 mM MgCl_2_, 0.1% NP-40, 0.1% Tween 20, and 0.01% digitonin] and incubated on ice for 3 min. A total of 1 ml of cold wash buffer [10 mM tris-HCl (pH 7.4), 10 mM NaCl, 3 mM MgCl_2_, and 0.1% Tween 20] was then added to the lysis. The sample was centrifuged at 500*g* for 10 min at 4°C. The pelleted nuclei were resuspended in 50 μl of transposition mixture [25 μl of 2× Tagment DNA buffer, 5 μl of Tagment DNA Enzyme (Nextera Sample Prep Kit from Illumina, FC-121-1030), 16.5 ml of PBS, 0.5 ml of 0.1% digitonin, 0.5 ml of 10% Tween 20, and 2.5 ml of H_2_O] and incubated at 37°C for 2 hours in a ThermoMixer with 1000 rpm mixing. Transposed DNA was purified with the MinElute Reaction Cleanup Kit (QIAGEN, 28204) and eluted in 21 μl of water. Purified DNA was amplified using 25 μl of the NEBNext High Fidelity 2x PCR Master Mix, 1.25 mM Nextera custom primers with unique barcodes, and nuclease-free water. Samples were amplified using the following PCR conditions: 72°C for 5 min; 98°C for 30 s; and cycled at 98°C for 10 s, 63°C for 30 s, and 72°C for 1 min. Half of each sample was amplified for 12 cycles, purified, and assessed by Bioanalyzer (Agilent) for library quality. Sample concentration was quantified by Qubit (Invitrogen) before pooling. Pooled samples were sequenced on a NextSeq 500/550 at the Genomics Core of Gladstone Institutes (paired-end, 75 bp, >80 million reads per sample).

### Analysis of genome sequencing data (ChIP-seq, RNA-seq, and ATAC-seq)

#### Genomic coordinates and gene annotation

All analysis was performed using RefSeq (GRCm38/mm10) gene annotations.

#### RNA-seq data processing and gene expression quantification

RNA-seq was aligned to the mm10 genome using STAR (version STAR_2.5.1b) (*62*) with default parameters. Transcript expression quantification was performed using featureCounts (version 1.6.3) (*63*) with default parameters to generate gene expression values in units of reads per kilobase per million mapped reads (RPKM).

#### Calculating read density

We calculated the normalized read density of a ChIP-seq dataset in any genomic region using the Bamliquidator (version 1.0) read density calculator (https://github.com/BradnerLab/pipeline/wiki/bamliquidator). Briefly, ChIP-seq reads aligning to the region were extended by 200 bp, and the density of reads per base pair was calculated. The density of reads in each region was normalized to the total number of million mapped reads producing read density in units of reads per million mapped reads per base pair (rpm/bp).

#### Differential gene expression analysis

Differential gene expression analysis was conducted using the edgeR package (*64*). Nonexpressed genes (<1 RPKM expression in all samples) were filtered out. Samples were normalized by effective library size, common and tagwise dispersions were estimated with default methods, and the exact test option was used to determine differential expression between control and KLF15-LKO conditions. False discovery rate (FDR) correction was done by the Benjamini-Hochberg method. Genes were considered significantly down-regulated if they had an FDR of <0.05 and log_2_ fold change of ≤−1 and were considered up-regulated if they had an FDR of <0.05 and log_2_ fold change of ≥1.

#### Creating heatmap of gene expression

Microarray gene expression profiles from *Klf15*^−/−^ mouse liver tissue have been published by Gray *et al.* (*16*) and were downloaded from Gene Expression Omnibus (GEO) (GSE7137). The top 20 significantly down-regulated genes in *Klf15*^−/−^ mouse liver tissue were identified, and the *z* score of expression levels (average of all probes for a gene) for each sample was visualized as a heatmap.

#### GSEA of RNA-seq data

GSEA (*65*) analysis was conducted on all expressed genes using default parameters and log_2_ ratio of classes as the metric for ranking genes. The hallmark gene sets were used for leading edge enrichment analysis, and enriched gene sets (FDR < 0.05) for each condition were visualized as a bar graph.

#### ChIP-seq data processing

KLF15, HN4a (GEO, GSE77670), and GR/NR3C1 (GEO, GSE108689) ChIP-seq data were processed using the AQUAS Transcription Factor and Histone ChIP-seq processing pipeline (https://github.com/kundajelab/chipseq_pipeline). Briefly, fastq files were aligned to the mm10 genome using BWA (*66*) with default pipeline parameters. Bam file deduplication and cross-correlation analysis were done according to ENCODE Consortium standards (*67*). Peak calls were done with the MACS2 peak finding algorithm (*68*) at a significance level of *P* < 0.01, and irreproducibility discovery rate (https://projecteuclid.org/euclid.aoas/1318514284) thresholded peaks were used as the final peak set. H3K27ac, H3K4me3, RNA Pol II, H3K4me1, and H3K27me3 ChIP-seq were obtained directly from the ENCODE database (identifiers: ENCSR000CNI, ENCSR000CEN, ENCSR000CBR, ENCSR000CAX, ENCSR000CA, and ENCSR000CAO) (*69*).

#### ATAC-seq data processing

ATAC-seq data were processed using the Kundaje laboratory ATAC-seq/DNase-seq pipeline (https://github.com/kundajelab/atac_dnase_pipelines). Data were processed as described for ChIP-seq, with the addition of TN5 shifting of reads following read filtering and deduplication.

#### Creating heatmap and meta representations of ChIP-seq occupancy

Heatmaps and meta plots of ChIP-seq occupancy for various factors were created as in the work of Lin *et al.* (*70*). Heatmaps were created for KLF15 and HN4a peaks and up- and down-regulated gene sets. Each row plots the ±5-kb region flanking the peak center with rows ordered by peak signal in the case of transcription factors. For gene sets, each row plots the ±5-kb region flanking the TSS of the genes, and rows are ordered by the sum of KLF15 signal in the region.

#### Annotating ChIP-seq peaks

KLF15 peaks were defined as promoter peaks if they occurred within ±2 kb from a known TSS and defined as enhancer peaks if they overlapped a region of H3K27ac and were not categorized as promoter regions. CpG islands were downloaded from the UCSC Genome Browser (*71*), and overlap was determined with bedtools intersect (*72*).

#### Determining distributions of peak distances to nearest genes

For each transcription factor (KLF15 and HNF4a), we measured the distance (within 1 Mb) to the nearest TSS (as previously defined), and the results were visualized as a histogram. Statistical comparison of distributions was done using the Epps-Singleton test.

#### Gene ontology term enrichment analysis

The closest genes (within 1 Mb) for each KLF15 peak were identified, and gene ontology enrichment analysis for gene ontology biological process terms was conducted using the PANTHER overrepresentation analysis (*73*) with Fisher’s exact test. Terms were summarized using REVIGO (*74*), and the top terms (FDR < 0.05) were visualized by a bar graph.

#### De novo motif analysis

De novo motif enrichment was carried out using MEME-ChIP (*75*), with input genomic regions defined as ±500 bp from the center of all KLF15 peaks. SP1 motif was obtained from the JASPAR database (*76*).

#### Computing gene-level KLF15 promoter signal

Promoter KLF15 signal was defined as the sum of the background signal (whole-cell extract)–subtracted area under the curve (AUC) of all KLF15 peaks within the promoter (±2 kb around TSS) of a gene. The statistical significance of changes in distributions among up-regulated, down-regulated, and nondifferentially expressed genes was assessed using a Mann-Whitney *U* test.

#### Bioinformatic identification of KLF15 interactome using STRING database

Functional and physical protein associations were obtained from the STRING database with medium confidence level (0.4) and interactions from both experimental sources and computational predictions. Both first and second shell interaction partners were captured.

#### Comparing KLF15 or NR3C1/GR occupancy signal to changes in gene expression levels

Expressed genes were identified as above and binned by KLF15 promoter signal or proximal GR/NR3C1 signal to generate a waterfall plot. The log_2_ fold change of gene expression level in each bin was plotted, along with a trend line of the median log_2_ fold changes in each bin.

#### Quantifying proximal and distal transcription factor occupancy for expressed genes

Proximal (promoter) KLF15 signal was computed as described above. Distal HNF4a signal is defined as the sum of the background-subtracted AUC for all HNF4a peaks within 1 Mb of a gene and not within the promoter region (±2 kb).

#### Determining leading edge enrichment for KLF15-associated genes

ATAC-seq signal was defined as the sum of the AUC of all ATAC-seq peaks within the promoter (±2 kb around TSS) of a gene. Log_2_ fold change of control signal over KLF15-LKO signal was calculated for each gene and used to rank genes by change in ATAC-seq. Using GSEA (*65*), we determined the leading edge enrichment score for the set of down-regulated genes with KLF15 promoter peaks.

#### Rank analysis of ATAC-seq peaks

ATAC-seq peak sets under control and KLF15-LKO conditions were merged, and peaks were ranked in ascending order by their mean AUC (*n* = 2) for a given condition. Peaks within the promoter region were selected, and control rank versus KLF15-LKO was visualized as a scatterplot. Points were colored by KLF15 AUC in the open chromatin region defined by the ATAC-seq peak.

#### Clustering analysis of transcription factor motifs with KLF15 peaks

Motif co-occurrence with KLF15 peaks was generated by first filtering for actively expressed transcription factors with a known motif in the HOCOMOCO v11 core mouse transcription factor database (*77*). Deoxyribonuclease (DNase) hypersensitivity peaks in mouse liver were obtained from ENCODE to define regions of open chromatin, and FIMO (*78*) was used to find the occurrence of transcription factor motifs within each peak. DNase hypersensitivity peaks were also annotated for overlap with KLF15 peaks. A distance matrix for each transcription factor pair was determined using correlation as a metric, and this distance matrix was hierarchically clustered on the basis of Euclidean distance.

## Notes

View/request a protocol for this paper from *Bio-protocol*.
