# Supplementary material for: KLF15 cistromes reveal a hepatocyte pathway governing plasma corticosteroid transport and systemic inflammation
Source: Sci Adv. 2022 Mar 9;8(10):eabj2917. doi: 10.1126/sciadv.abj2917 (PMC8906731; doi:10.1126/sciadv.abj2917)
Supplement: Supplementary file 1 — Figs. S1 to S7 Tables S2 and S3 [file sciadv.abj2917_sm.pdf]

Science Advances
AAAS

Supplementary Materials for

KLF15 cistromes reveal a hepatocyte pathway governing plasma corticosteroid transport and systemic inflammation

Zhen Jiang, Selma Z. Elsarrag, Qiming Duan, Edward L. LaGory, Zhe Wang, Michael Alexanian, Sarah McMahon, Ingrid C. Rulifson, Sarah Winchester, Yi Wang, Christian Vaisse, Jonathan D. Brown, Mattia Quattrocelli, Charles Y. Lin*, Saptarsi M. Haldar*

*Corresponding author. Email: shalda01@amgen.com (S.M.H.); charles.y.lin@bcm.edu (C.Y.L.)

Published 9 March 2022, *Sci. Adv.* **8**, eabj2917 (2022)
DOI: 10.1126/sciadv.abj2917

The PDF file includes:

Figs. S1 to S7
Legend for table S1
Tables S2 and S3

Other Supplementary Material for this manuscript includes the following:

Table S1

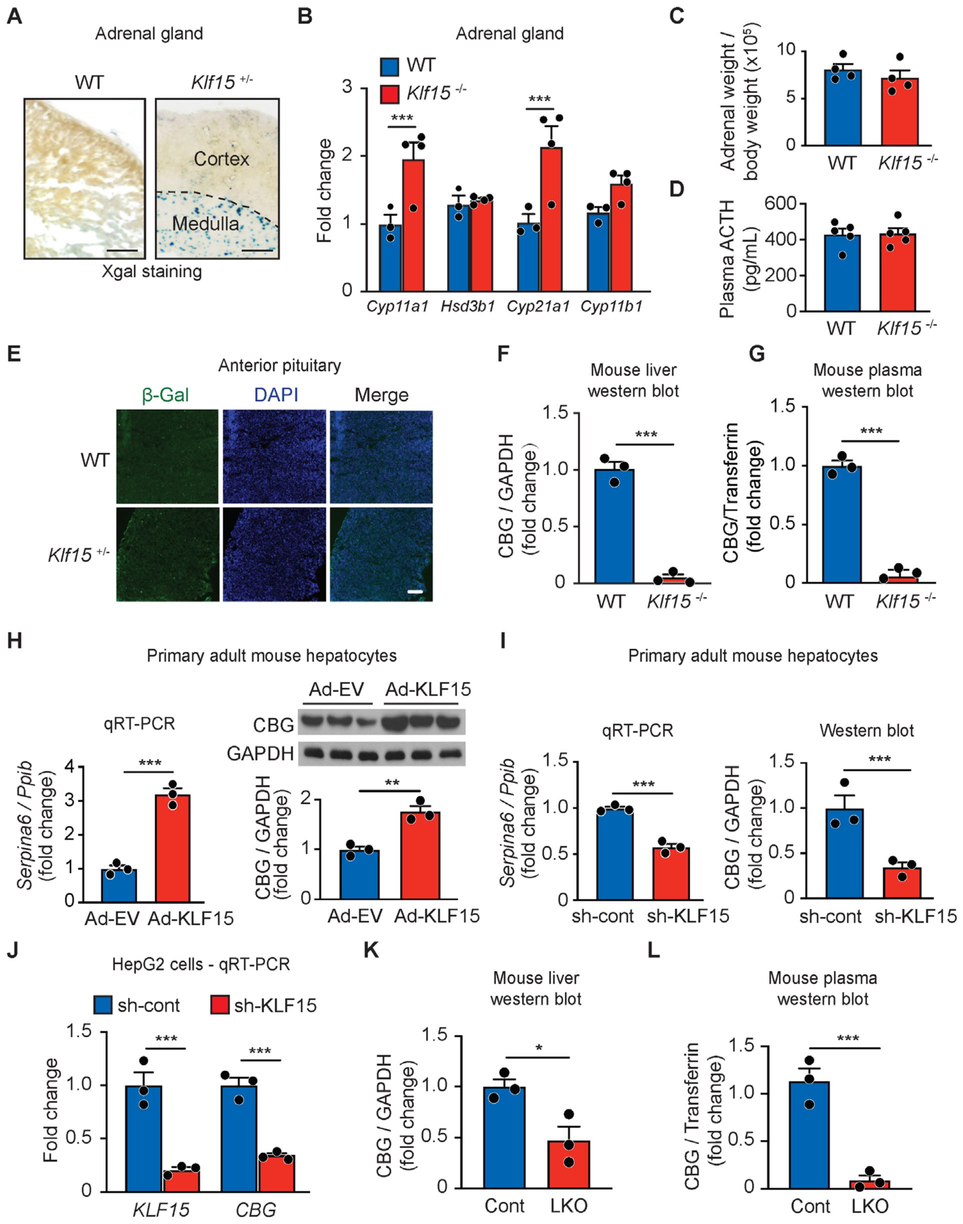

Fig. S1. Hepatocyte KLF15 potently regulates SERPINA6/CBG expression and plasma corticosteroid binding capacity. (A) Representative images of X-gal stained adult mouse adrenal gland from *Klf15*$^{+/-}$ vs. *Klf15*$^{+/+}$ mice. The null allele has a nuclear-localized lacZ expression cassette inserted into exon 2 of the native mouse *Klf15* locus. There is no detectable LacZ staining (reflecting *Klf15* expression) in the adrenal cortex. Cells within the adrenal medulla are positive

(demarcation between adrenal cortex and medulla shown by hashed line). Scale bar = 100 um. **(B)** qRT-PCR from mouse adrenal gland tissue from *Klf15*$^{+/+}$ vs. *Klf15*$^{+/+}$ mice for key genes in adrenal corticosteroid biosynthetic pathway (N=3-4). Data normalized to *Ppib*. **(C)** Ratio of adrenal gland weight to body weight (N=4) and **(D)** plasma ACTH concentration (N=5) in *Klf15*$^{+/-}$ vs. *Klf15*$^{+/+}$ mice. **(E)** Immunofluorescence staining for beta-galactosidase (β-gal) protein in anterior pituitary gland from *Klf15*$^{+/-}$ vs. *Klf15*$^{+/+}$ mice. There is no detectable beta-galactosidase expression (reflecting *Klf15* expression) in the anterior putuitary. Scale bar = 100 um. **(F)** Western blot quantification of CBG protein abundance in liver tissue (N=3) and **(G)** plasma (N=3) from *Klf15*$^{+/-}$ vs. *Klf15*$^{+/+}$ mice. **(H)** Right panel: qRT-PCR of *Serpina6* mRNA expression (normalized to *Ppib*) in wild type mouse primary hepatocytes infected with adenovirus for KLF15 overexpression (Ad-KLF15) or empty vector (EV) control. N=3. Left panel: Western blot of CBG and GAPDH (loading control) protein in Ad-KLF15 and EV infected wild type mouse primary hepatocytes (top) with quantification below (N=3). **(I)** qRT-PCR of *Serpina6* mRNA (normalized to *Ppib*) (left) and Western blot quantification (right) in wild type mouse primary hepatocytes infected with adenovirus expressing KLF15 shRNA (sh-KLF15) or scrambled shRNA control (sh-cont). N=3. **(J)** qRT-PCR of human *KLF15* and *SERPINA6* mRNA in HepG2 human hepatoma cell line infected with Adenovirus expressing KLF15 shRNA (sh-KLF15) or scrambled shRNA control (sh-scrambled). N=3. Data normalized to *PPIB*. **(K)** Quantification of western blot for CBG protein abundance in liver tissue and **(L)** plasma from of KLF15-LKO vs control mice. N=3. For all panels in Fig. S1: bars indicate mean $\pm$ SEM. *P<0.05, **P<0.02; ***P<0.01 by two-tailed, unpaired *t*-test.

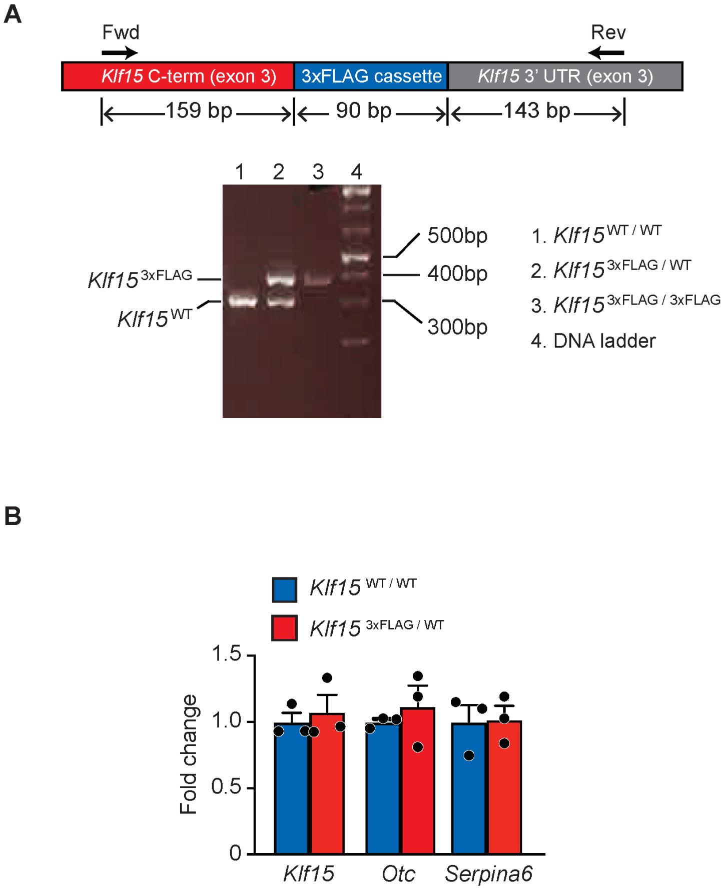

Fig. S2. Validation of a newly generated *Klf15*^{3xFLAG} knock-in mouse line. (A) PCR of genomic DNA from adult mice demonstrating germline transmission of the *Klf15*^{3xFLAG} allele and viability of mice heterozygous and homozygous for the *Klf15*^{3xFLAG} allele. Locations of PCR primers and amplicon sizes are schematized. **(B)** qRT-PCR of *Klf15* and known KLF targets (*Otc* and *Serpina6*) in liver tissue from adult *Klf15*$^{3xFLAG/WT}$ mice and *Klf15*$^{WT/WT}$ control mice reveals no significant differences in gene expression between genotypes (N=3; using two-tailed, unpaired *t*-test). Data normalized to *Ppib*. Bars indicate mean $\pm$ SEM.

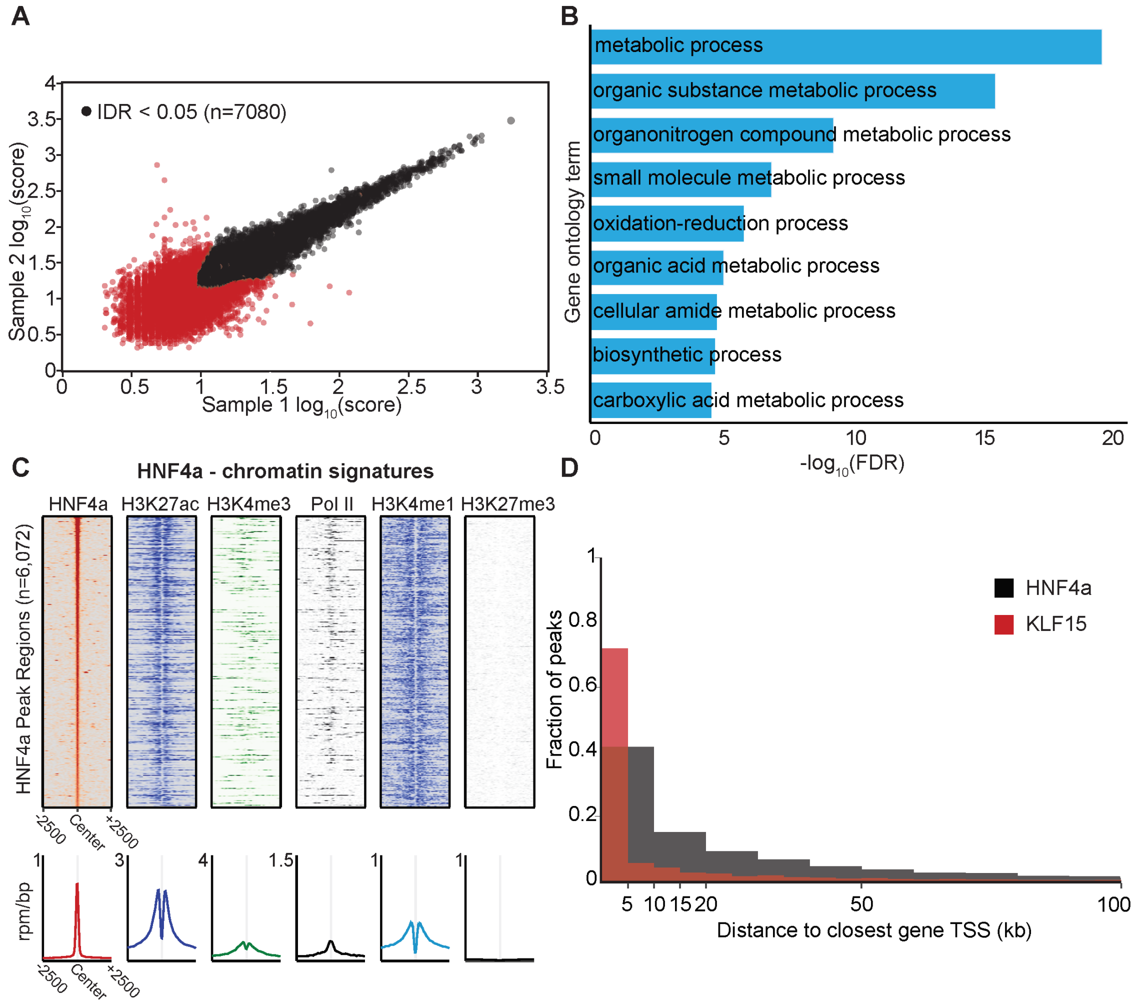

Fig. S3. Analysis of KLF15 binding sites in adult mouse liver reveals that it is predominantly promter-bound. **(A)** KLF15 replicate-1 log_{10} peak scores versus replicate-2 log_{10} peak scores. Peaks below IDR threshold of 0.05 (n=7,080) in black. **(B)** Top gene ontology terms (biological process) associated with genes with KLF15 promoter peaks. **(C)** Heatmaps of HNF4a, H3K27ac, H3K4me3, RNA Polymerase II (Pol II), H3K4me1, and H3K27ac signal (rpm/bp) along 5kb regions centered at HNF4a peaks. Regions are ordered based on HNF4a transcription factor peak signal (highest to lowest). Average signal profile (rpm/bp) for histone modification or transcription

factor plotted below each heatmap. **(D)** Histogram of fraction of KLF15 (red) and HNF4a (black) peaks in each bin (y axis) that fall within a given distance (kb) to a gene transcription start site (x axis). Distributions are significantly difference ($P < 0.001$, Epps-Singleton test).

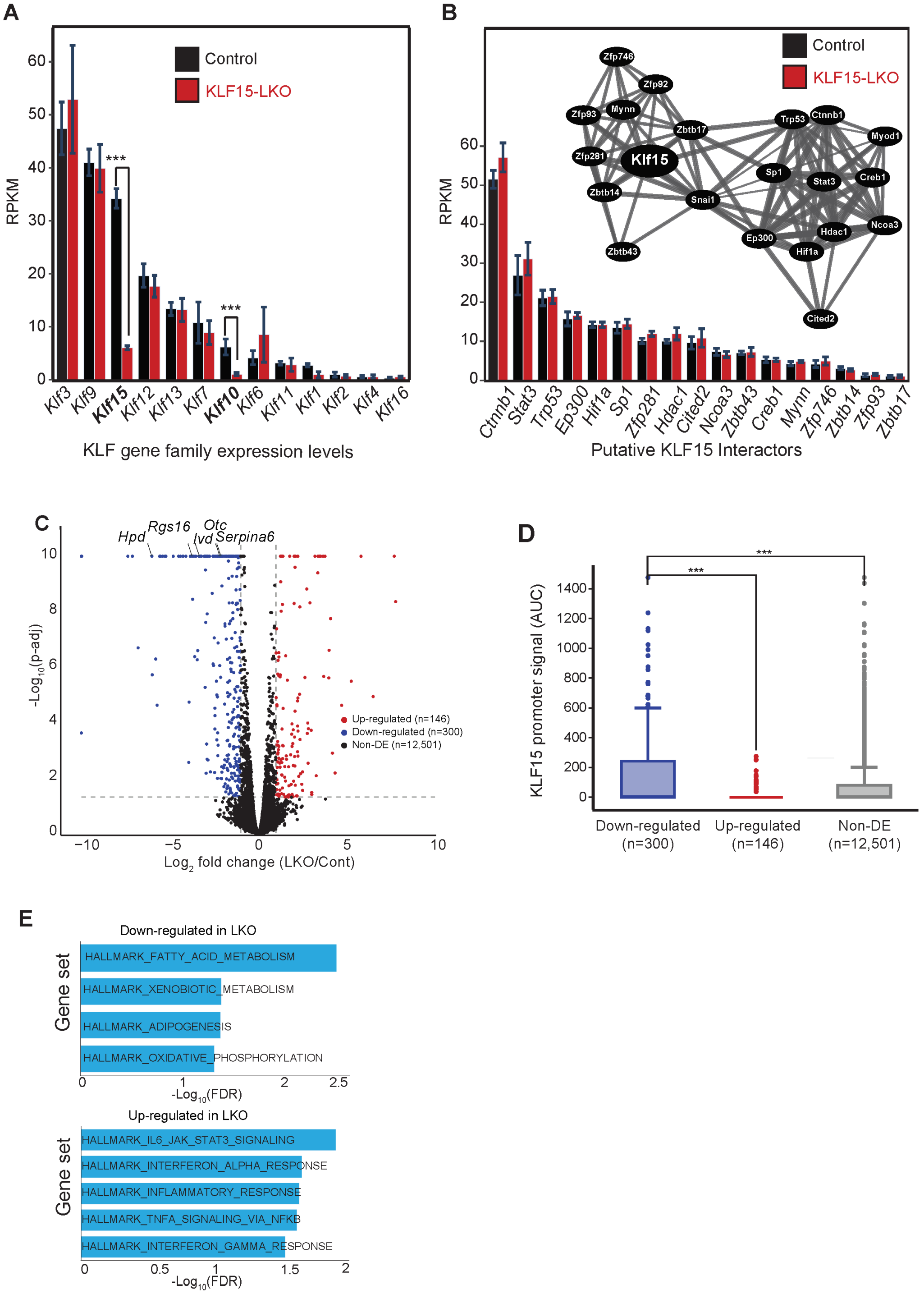

Fig. S4. Liver KLF15 is predominantly promoter-bound and functions as a transactivator of its directly bound targets. (A) Bar plot showing expression in units of RPKM of KLF-family of

transcription factors in control mouse livers (black) and KLF15-LKO livers (red). Error bars represent standard deviation (N=4 replicates). Statistical significance of the difference between control and KLF15-LKO assessed by a two-tailed, unpaired t-test. *** $p < 10^{-9}$. (**B**) Bar plot of expression in units of RPKM for KLF15 interacting proteins (N=4) as defined by the STRING protein-protein interaction database (inset). **(C)** Volcano plot showing the $\log_2$ fold change of gene expression (x axis) and false discovery rate (FDR)-adjusted p values (y axis) for all expressed genes. The dotted line represents a 0.01 FDR cutoff. **(D)** Box plot of total KLF15 peak signal (AUC) within promoters of genes down-regulated, up-regulated, and non-differentially expressed in livers of KLF15-LKO mice. ***$P < 0.001$ by Mann-Whitney U test. **(E)** Significantly enriched hallmarks gene-sets in liver tissue from control mice (top) and KLF15-LKO mice (bottom) discovered by GSEA.

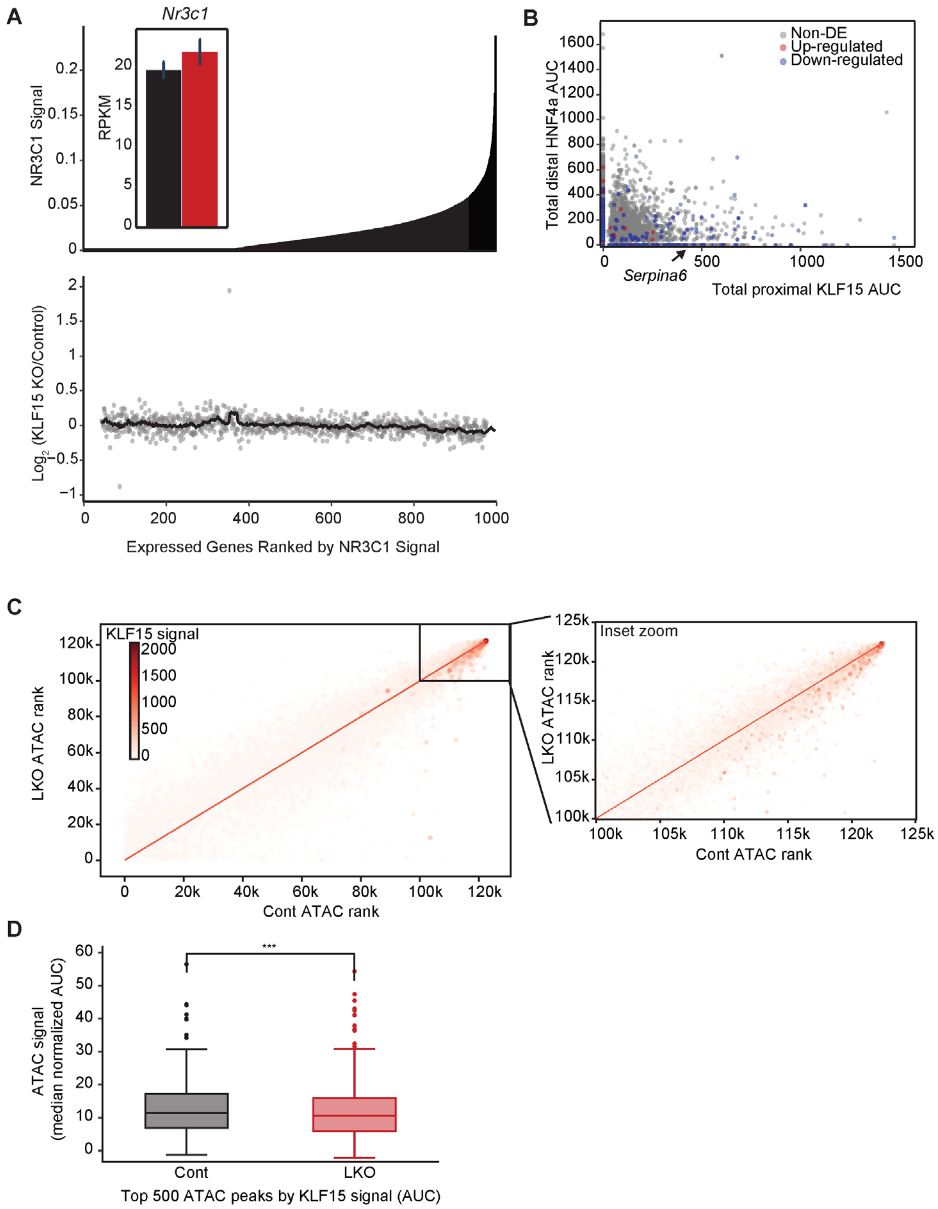

Fig. S5. DNA-bound KLF15 is required to maintain local open chromatin in the liver. (A) Upper panel: median glucocorticoid receptor (NR3C1) ChIP-seq signal over transcription start sites for expressed genes sub-divided into 1,000 bins and rank ordered by KLF15 TSS signal. Upper panel inset: Bar plot of *Nr3c1* mRNA levels in control (black) and KLF15-LKO (red) mouse livers. Error bars represent standard deviation (N=4 replicates). Lower panel: median $\log_2$ fold

change (KLF15-LKO/control) for genes within each bin. **(B)** Scatter plot of total KLF15 peak AUC within gene promoters vs. total HNF4a peak AUC at distal sites (within 50kb) of gene TSS for all expressed genes. Genes downregulated in KLF15-LKO colored in blue, and genes upregulated in KLF15-LKO colored in red**.** **(C)** Left panel: scatter plot of ranks for promoter associated ATAC-seq peaks in control livers vs KLF15-LKO livers. Peaks are ranked by AUC (ascending order) for genome-wide set of consensus peaks across control and KLF15 knock out conditions. Line represents points where rank is unchanged in KLF15-LKO condition from control. Points are colored by KLF15 signal within ATAC-seq regions. Inset callout panel: top ranked peaks (n=22,588) from upper panel. **(D)** Box plot of median normalized ATAC-seq signal (AUC) for top 500 ATAC-seq peaks by KLF15 signal in control and KLF15-LKO conditions. ***P<0.001 by Wilcoxon signed-rank test.

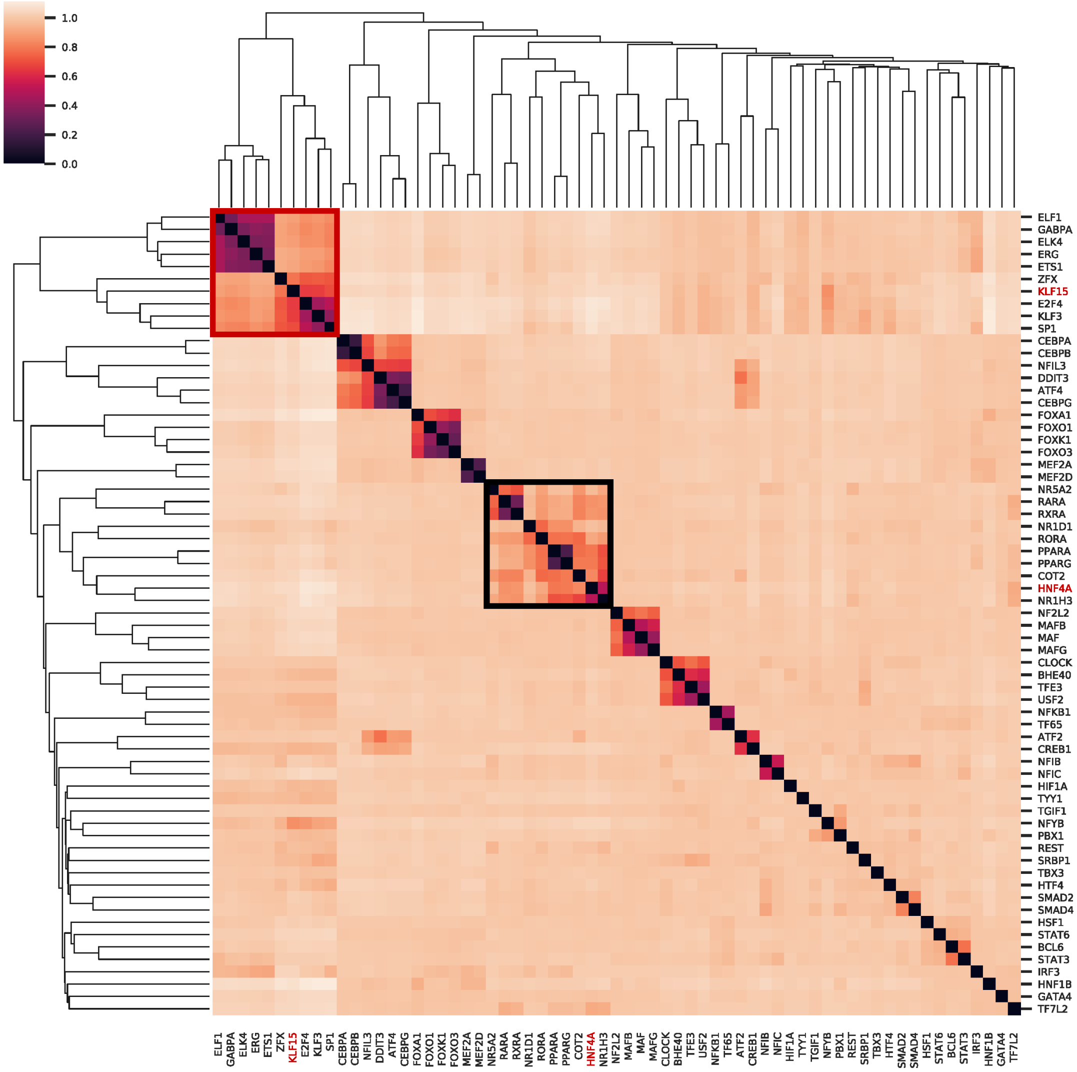

Fig. S6. Clustering of KLF15 binding sites with other transcription factor binding motifs. Clustergram heatmap showing pairwise similarities between occurrence of detectable motif within a region of open chromatin for all expressed transcription factors with known motifs. KLF15 occurrences defined by presence of a KLF15 peak within open chromatin region. Pairwise distances (1-Pearson correlation) are shaded from purple to yellow. Promoter associated factors are indicated by the red box and liver enhancer associated factors are indicated by the black box.

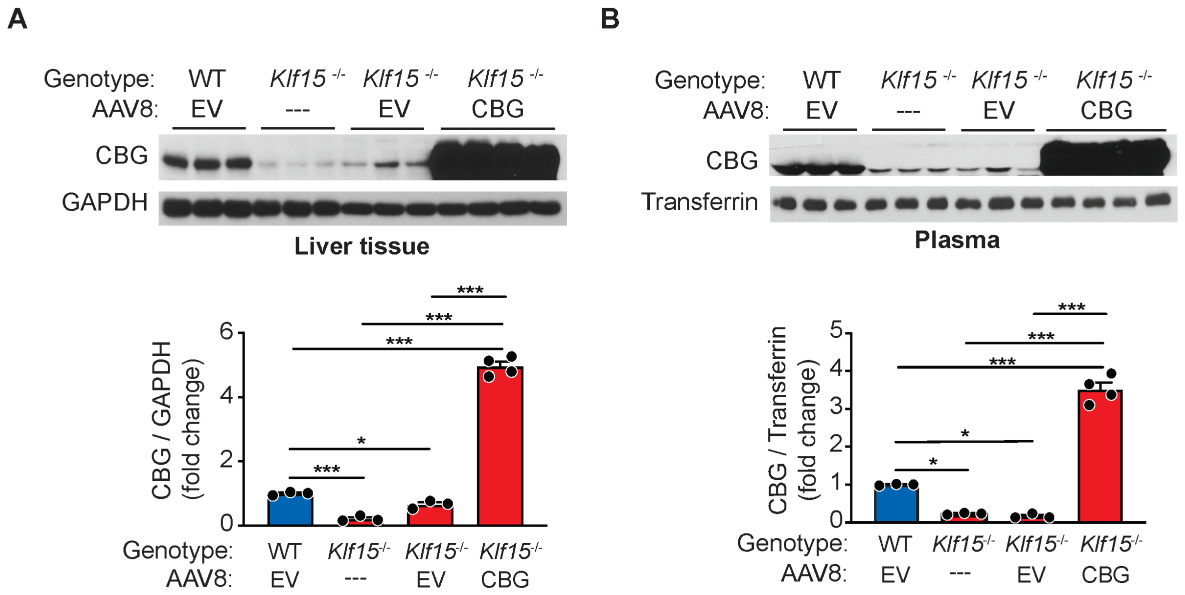

Fig. S7. AAV8-mediated rescue of CBG expression and function in KLF15-deficient mice. **(A)** Western blot for CBG and GAPDH (loading control) protein abundance in liver tissue from mice of indicated genotypes, six weeks after tail vein injection with AAV8-CBG or empty vector (EV) control. Quantification of CBG abundance is below (N=3-4). **(B)** Western blot for CBG and Transferrin (loading control) protein abundance in plasma from mice of indicated genotypes, six weeks after tail vein injection with AAV8-CBG or empty vector (EV) control. Quantification of CBG abundance is below (N=3-4). For panels b-c, an extra control from *Klf15*$^{-/-}$ liver tissue or plasma was included during the in vivo AAV8 validation to assess whether the empty vector (EV) had any significant effect on CBG abundance; *P<0.05, **P<0.02, ***P<0.01 by two-way ANOVA followed by Tukey's multiple comparison test. For all panels, data shown as mean $\pm$ SEM.

Table S1. Output of STRING database of KLF15 interacting proteins. Data provided online as a separate Excel spreadsheet file.

Table S2. Plasma cytokine profiles in Control and KLF15-LKO mice 24 hours after LPS injection. Mean values are shown. *P*-values for effect of KLF15-LKO genotype status on LPS response was determined using two-way ANOVA.

	Saline		LPS		
**Plasma Cytokine**	**Control (N=13)**	**LKO (N=11)**	**Control (N=17)**	**LKO (N=11)**	***P*-value; KLF15-LKO effect on cytokine concentration after LPS**
**Eotaxin**	430.6	277.1	2897	3029	0.818
**G-CSF**	384.6	367.4	41114	41365	0.311
**GM-CSF**	21.28	13.53	42.77	37.78	0.269
**IFNγ**	1.495	5.015	1969	2175	0.953
**IL-1a**	31.88	4.044	578.3	851	<.001
**IL-1B**	4.187	3.557	262.2	1050	<.001
**IL-2**	5.6	4.684	11.16	9.522	0.204
**IL-3**	1.246	0.7344	2.141	1.878	0.388
**IL-4**	1.291	0.294	0.585	0.4525	0.911
**IL-5**	12.09	9.946	84.62	120.7	<.001
**IL-6**	4.573	4.38	25589	26207	0.019
**IL-7**	14.09	39.75	6.123	4.638	0.844
**IL-9**	19.07	21.11	28.07	37.95	0.011
**IL-10**	11.58	6.894	540.7	1705	<.001
**IL-12 (p40)**	17.19	10.31	76.79	53.34	0.095
**IL-12 (p70)**	77.86	29.9	87.88	70.98	0.865
**IL-13**	19.94	20.25	126.5	122.9	0.745
**IL-15**	69.43	162.6	133	145.6	0.959
**IL-17**	6.965	3.284	4587	4951	0.624
**IP-10**	80.48	103	13887	21733	0.059
**KC**	79.56	136.1	26117	26265	0.91
**LIF**	1.912	4.987	1275	340.3	<.001
**LIX**	1403	378.4	2146	2445	0.582
**MCP-1**	71.87	48.79	26390	26900	0.876
**M-CSF**	8.266	7.516	664.5	584.4	0.625
**MIG**	17.04	32.82	1593	1671	0.546
**MIP-1a**	61.55	55.42	386.3	577.2	0.003
**MIP-1B**	54.53	62.5	3677	5954	0.013
**MIP-2**	108.5	90.73	16275	17512	0.703
**RANTES**	36.52	17.21	1682	1740	0.927

**TNFa**	11	7.894	282.1	248.9	0.076
**VEGF**	0.5793	0.72	9.211	6.062	0.041

Table S3. qRT-PCR primer and probe information

Gene	Species	Forward Primer	Reverse Primer	Roche Universal Probe Library TaqMan Probe No.
Serpina6	Mouse	ccaccaaagacactcccttg	ggtgtacaggagggccatt	#40
Klf15	Mouse	acaggcgagaagccctttt	catctgagcgggaaaacct	#64
Ppib	Mouse	ttcttcataaccacagtcaagacc	accttccgtaccacatccat	#20
Cyp11a1	Mouse	aggccaacattaccgagatg	ggttccactgcagggtcat	#51
Hsd3b1	Mouse	gaccagaaaccaaggaggaa	gcactgggcatccagaat	#12
Cyp21a1	Mouse	cgagaccacggctaccac	actcttcctgcagtcgcttc	#26
Cyp11b1	Mouse	agctcagacttggtgcttcag	gcccatggaatacagattcac	#3
SERPINA6	Human	ggacacgattaacaggtggtc	gagatggtgacctttggaatg	#66
KLF15	Human	caaaagcagccacctcaag	ggtacggcttcacacctga	#19
GAPDH	Human	agccacatcgctcagacac	gcccaatacgaccaaatcc	#60
